# Equity premium forecasting with reliability-screened forward-looking signals

**DOI:** 10.1371/journal.pone.0341578

**Published:** 2026-05-15

**Authors:** Jeonggyu Huh, Jaegi Jeon, Seungwon Jeong

**Affiliations:** 1 Department of Mathematics, Sungkyunkwan University, Suwon, Republic of Korea; 2 Graduate School of Data Science, Chonnam National University, Gwangju, Republic of Korea; 3 Global-Learning & Academic research institution for Master’s· PhD students, Chonnam National University, Gwangju, Republic of Korea; Incheon National University, KOREA, REPUBLIC OF

## Abstract

Forecasting the equity risk premium is challenging because predictive relationships are unstable out of sample. We propose a two-stage framework that generates forward-looking signals from standard macro-financial predictors and admits them only when they satisfy predictor-level reliability criteria. In Stage 1, each predictor is forecast one step ahead to obtain an expected movement and an uncertainty proxy. In Stage 2, lagged predictors are augmented with the admitted signals and mapped into next-period excess returns using random forests, optionally combined with SHAP-guided screening and dimension reduction. Using monthly U.S. data from 1952 to 2024, we evaluate both benchmark-relative out-of-sample accuracy and the economic value of forecasts in a constrained mean-variance allocation. Selective admission improves out-of-sample accuracy and, in selected specifications, yields economically meaningful gains in risk-adjusted performance and drawdown control. Tail-conditional diagnostics show that these gains are concentrated disproportionately in downside market states, helping explain when statistical improvements translate into economic value. The main qualitative patterns remain robust across alternative learners, across the S&P 500 and the CRSP value-weighted market index, and under net of transaction cost evaluation and alternative portfolio volatility checks. Taken together, the findings suggest that forward-looking predictor information is most useful when admitted selectively on the basis of predictor-level reliability, with its practical value lying less in universal forecast improvements than in more robust and downside-sensitive equity premium forecasting.

## 1 Introduction

Whether the equity risk premium can be forecast in real time remains a central question in empirical finance and a practical concern for portfolio allocation. A long tradition documents in-sample links between macro-financial predictors and subsequent market returns, yet robust out-of-sample improvements over simple benchmarks are notoriously difficult to obtain and often unstable across samples and evaluation designs [1,2]. At the same time, even modest forecasting gains can be economically meaningful for mean-variance investors because portfolio weights scale with the predicted premium relative to risk [3]. This tension—statistical fragility versus potential economic value—motivates renewed attention not only to forecasting accuracy itself, but also to how predictive information should be engineered and disciplined under realistic real-time data constraints. More fundamentally, equity premium forecasting is a demanding prediction problem: Realized returns are noisy, the conditioning set can be high dimensional, predictive relationships may shift across regimes, and the relevant interactions among predictors may be nonlinear. Recent work increasingly approaches such environments using advanced machine learning and time-series econometric tools designed to handle instability, nonlinearities, and high-dimensional inputs.

A key inspiration for our approach comes from the factor timing literature, which emphasizes that expected returns vary over time and that exploiting this variation can matter for investors [4–8]. In practice, however, strategies that attempt to forecast the equity premium directly face a difficult signal-extraction problem. Rather than applying a highly flexible end-to-end learner directly to noisy realized returns, we take a modular detour: We first forecast the predictor panel itself—variables observed at the forecast origin that often evolve more smoothly than realized returns—and then use those forecasts to construct forward-looking inputs for equity premium prediction. The key intuition is that the current level of a predictor need not fully summarize the state relevant for next period premia. Two episodes with similar observed predictor-levels can imply different expected returns if the predictor is expected to deteriorate further, stabilize, or reverse, and if that transition is more or less uncertain. In this sense, robust equity premium forecasting may require not only past predictor-levels, but also disciplined forward-looking information about where those predictors are likely to move and how reliable those implied movements are.

This *predict-the-predictors* perspective connects to a complementary strand that constructs forward-looking state variables by embedding expectations about future cash flows or risks into signals observed today. Prominent examples include implied expected return measures inferred from market prices and cash-flow forecasts [9,10], as well as option implied measures that extract forward-looking information about tail risk and the risk neutral distribution [11,12]. Our notion of forward-looking differs in both object and construction. We do not rely on derivatives or survey data, nor do we propose a new implied premium series. Instead, we generate forward-looking signals internally from the standard macro-financial predictor panel and ask whether they can improve real-time equity premium forecasting when they are admitted only after clearing a predictor-level out-of-sample reliability screen.

In this study, we propose a two-stage framework that converts the standard macro–financial predictor panel into forward-looking features for equity premium forecasting. In Stage 1, each predictor is forecast one-step-ahead to form a forward-looking pair capturing its expected movement and a proxy for forecast uncertainty. These generated signals are admitted into the combined feature set only when they clear a predictor-level out-of-sample reliability threshold. In Stage 2, we augment lagged predictor levels with the admitted forward-looking pairs, optionally apply SHAP-based pre-screening for parsimony, reduce dimension via PCA or PLS, and learn a nonlinear forecasting rule for the next-period equity premium using a random forest [13] as the baseline learner. In implementation, we optionally apply Shapley (SHAP) value importance based screening for parsimony and use PCA or PLS for dimension reduction. We implement the pipeline under real-time availability constraints and apply it to monthly U.S. data spanning 1952–2024 using a standard predictor set from the seminal works of [1] and [2], with the S&P 500 as the baseline market and, as a cross-index robustness check, the CRSP value-weighted index evaluated under the same training, validation, and test windows as the baseline S&P 500 analysis. To check that the conclusions are not tied to a single Stage 2 learner, we also report supplementary robustness exercises using XGBoost and LightGBM.

We evaluate statistical predictability using benchmark-relative out-of-sample *R*^2^ and tail-conditional counterparts that distinguish downside from upside accuracy, and we complement these with formal forecast-comparison tests against the historical mean benchmark and against matched past-only designs. Economic value is assessed by mapping forecasts into a simple constrained mean-variance allocation following [3] and by examining the resulting portfolio performance net of transaction costs. Empirically, the gains from forward-looking augmentation are selective rather than uniform: Support is more evident in benchmark-relative comparisons than in stricter head-to-head tests between otherwise matched specifications, where one model uses only lagged predictor-levels and the other augments those same predictors with the generated forward-looking signals. The most economically relevant improvements arise when the forward-looking signals are admitted selectively rather than mechanically. These gains are especially visible in PLS-family representations, which more often concentrate predictive improvements in downside states and help explain why investment performance can improve even when average-fit gains remain modest. A sigma-ablation exercise further shows that the Stage 1 uncertainty proxy is not redundant and is most informative in downside-sensitive settings, while the XGBoost and LightGBM results broadly preserve the same qualitative pattern.

This paper makes three contributions. First, we propose a disciplined feature-construction pipeline that transforms standard macro-financial predictors into forward-looking signals—expected movements and uncertainty proxies—admits them through predictor-level real-time reliability screening, and uses SHAP-based pre-screening to build parsimonious forecasting sets while retaining an interpretable view of dominant predictor channels. Second, we evaluate the framework in a way that is explicitly state-dependent and economically implementable, combining tail-conditional diagnostics, formal forecast-comparison evidence, and portfolio results measured net of transaction costs. Third, we show that the clearest economically meaningful gains arise in downside-sensitive settings, especially in PLS-family representations, rather than as a blanket improvement in average forecast accuracy across all specifications.

The remainder of the paper is organized as follows. Section 2 reviews the related literature on equity premium predictability, forward-looking predictors, and machine learning based nonlinear forecasting. Section 3 presents the two-stage methodology and evaluation metrics. Section 4 describes the data, real-time implementation, and experimental design. Section 5 reports the main empirical results, covering Stage 1 forecastability, Stage 2 predictive accuracy, formal forecast-comparison evidence, portfolio performance net of transaction costs, and interpretability. Section 6 concludes with practical implications, limitations, and directions for future research.

## 2 Related literature

A long-standing literature examines whether aggregate excess stock returns are predictable using information available at the forecast origin. Classic studies consider linear predictive regressions with valuation ratios and term structure variables, including dividend-based measures and interest rate information [14–17]. This evidence is often interpreted through time variation in discount rates, and macro-finance state variables have been proposed to summarize such movements [18]. At the same time, predictability is well known to be fragile in finite samples and unstable across subsamples, motivating careful real-time evaluation and robustification strategies [1,19]. A prominent response emphasizes economically motivated restrictions and simple devices that can improve out-of-sample performance and avoid implausible forecasts [3], while another response aggregates information across many predictors via forecast combinations [20]. The predictor set has also been expanded beyond standard macro finance variables to include technical indicators that proxy for market trends and investor behavior [21]. Relatedly, [22] model market-trend dynamics by transforming momentum signals into probabilistic trend-transition features and using them as inputs to an LSTM-based trading model. Related time-series studies also examine whether auxiliary market information improves forecasts: Using rolling cointegration analysis and out-of-sample Granger-causality tests on Chinese stock-index and stock-index-futures prices, [23] finds that the two markets are generally cointegrated but that neither series consistently improves the other’s forecastability. In an agricultural price forecasting setting, [24] estimates models built from own-market history, nearby cash-market prices, and futures histories for U.S. corn buying locations, finding that nearby-market information improves forecasts with larger gains at longer horizons.

More recent work reframes conditional expected return estimation as a high dimensional prediction problem and applies statistical learning methods to capture nonlinearities and interactions among predictors. A central benchmark is [25], which systematically compares modern machine learning methods to traditional linear models in predicting and economically exploiting return variation. Related research moves beyond purely predictive performance toward economically structured representation learning: [26] proposes conditional autoencoder models that learn latent factors and nonlinear conditional exposures under asset pricing restrictions, thereby linking modern neural architectures to factor model interpretations. In a similar spirit, [27] develops deep learning architectures that explicitly separate time-series and cross-sectional components of risk premia and provides tools to interpret the resulting forecasts. Complementary work further advances deep learning tools for modeling nonlinear return predictability and conditional asset pricing relations, with an emphasis on disciplined out-of-sample validation in high dimensional settings [28,29].

Beyond the core equity premium literature, recent neural network studies apply relatively simple nonlinear architectures to Chinese rent indices, the Chinese energy security index, the China commodity price index, and U.S. corn cash prices, with the rent index, CCPI, and corn applications reporting stable and accurate forecasts and the corn study further showing that futures prices can improve one-step-ahead accuracy [30–33]. Gaussian process regression has likewise been used successfully in price forecasting, with Bayesian-optimized and cross-validated GPR models applied to daily coffee prices and ten Chinese steel product price indices [34,35]. Graphical techniques provide another route for modeling complex dependence patterns: VECM-DAG frameworks using the PC algorithm and LiNGAM have been employed to recover contemporaneous causal structures in Jiangsu housing markets and regional Chinese scrap-steel prices, revealing heterogeneous transmission channels and dominant nodes in the adjustment process [36,37]. Ensemble and composite methods offer a further complementary perspective, as combined-forecast frameworks improve U.S. corn cash price forecasting and shrinkage-based composite forecasts outperform individual time series models for the Chinese stock index across multiple horizons, underscoring the broader value of flexible forecasting architectures for capturing complex and potentially nonlinear dynamics [38,39].

A complementary literature constructs forward-looking predictors—objects observed at time *t* that embed beliefs about future cash flows, risks, or return distributions—and relates these signals to expected returns and realized excess returns. One prominent route backs out implied expected returns from market prices together with cash-flow forecasts, producing an implied equity premium series that can be used as a forward-looking state variable in empirical analysis [9,10]. Options markets provide another source of forward-looking information about risk compensation and return distributions; for example, option implied risk measures have been linked to subsequent market returns, and related work combines derivative implied information with time-series modeling to construct forward-looking market risk premium measures [11,12,40]. Relatedly, [41] show that economic policy uncertainty indices from ten developed countries contain out-of-sample predictive information for U.S. excess stock returns, with several non-U.S. EPU measures outperforming the domestic U.S. EPU index itself. From the perspective of predictor construction, these strands share a common message: rather than treating expected returns as an unobservable latent object, one can build practically usable forward-looking predictors by leveraging data sources that already embed market beliefs about the future.

Closest in spirit to our setting is a smaller branch that uses explicitly expectation driven or forecast generated regressors—direct measures of expected business conditions or professional macroeconomic forecasts—as inputs for studying expected returns and discount-rate variation. [42] connect directly measured expectations about business conditions to subsequent stock returns, highlighting the informational role of expectation variables beyond contemporaneous macro realizations. [43] examine what survey expectations imply for predictability in financial markets and how expectation data interact with standard predictability evidence. [44] use survey based beliefs about risk and return to link perceived economic conditions and expected premia, emphasizing that expectations themselves can act as economically meaningful predictors. [45] similarly exploit professional macroeconomic forecasts to construct expectation-based indices and relate them to expected returns, underscoring that explicitly forecast based inputs can play a distinct role relative to realized macro variables. Taken together, this literature motivates viewing forward-looking predictors not only as market implied objects from prices and derivatives, but also as generated expectation measures—an idea that naturally aligns with our focus on constructing forward-looking signals at the predictor-level and using them as inputs to risk premium forecasting models.

In empirical implementations, these forward-looking constructions naturally raise an additional practical question: how to screen and organize a large set of candidate regressors in a way that preserves out-of-sample performance while remaining interpretable. A recent explainable AI strand addresses this issue by leveraging SHAP not only for post hoc interpretation but also as a practical screening device for variable selection: covariates are ranked by aggregated Shapley attributions, a compact subset is retained, and the predictive model is refit on the reduced set to improve parsimony and robustness [46–48]. In financial forecasting, this SHAP-guided selection has begun to appear in end-to-end pipelines where the screened variables are explicitly reused for downstream prediction and decision-making. [49] selects a small set of top SHAP-ranked predictors from a broad feature pool, retrains a LightGBM return forecaster, and translates the resulting forecasts into a risk controlled, periodically rebalanced equity portfolio. [50] similarly use SHAP to identify the most influential inputs in a deep learning trend classifier and show that restricting the model to the top ranked features can preserve—and in some metrics improve—predictive performance while enhancing transparency. [51] goes further by integrating SHAP-based recursive feature elimination with hyperparameter optimization in an LSTM forecasting framework, aiming to obtain parsimonious yet accurate stock predictions. These pipelines primarily target cross-sectional stock return prediction and stock-selection strategies. By contrast, we use SHAP as an auxiliary screening and interpretation layer within a market level equity premium forecasting framework built on predictor generated forward-looking signals and tail-state diagnostics. Taken together, these studies motivate our use of SHAP importance based screening as a disciplined dimension-reduction step that aligns interpretability with predictive performance and with the economic evaluation of return and risk premium forecasts.

## 3 Methodology

We propose a two stage framework for forecasting the equity risk premium. Stage 1 forecasts each predictor to produce forward-looking signals; Stage 2 incorporates these signals into a machine learning forecasting model for the equity premium.

Our objective is to map the engineered features **X**_*t*_ into a risk premium forecast. Following [25], we write


$$\begin{array}{c} \begin{array}{c} r_{i,t+1}={𝔼}_{t}(r_{i,t+1})+\varepsilon_{i,t+1},\;{𝔼}_{t}(r_{i,t+1})=g({𝐗}_{t}), \end{array} \end{array}$$

where g(·) denotes the forecasting model used in this study; the specific model is described in Section 4.2.2. We now describe in detail how **X**_*t*_ is constructed.

### 3.1 Forecasting the predictors

We adopt a unified procedure that extracts forward-looking information from each predictor by producing a one-step-ahead conditional mean forecast and its one-step-ahead conditional residual variance as a measure of forecast uncertainty. We compute the variance to quantify the state-dependent precision of the mean forecast and carry these quantities forward to the next stage; how they are used is described later.

Let $\{z_{k,t}{\}}_{k=1}^{K}$ denote the *K* predictor series. For each predictor, we estimate a flexible ARIMAX ($p_{k}^{m},d_{k},q_{k}^{m}$) model, proposed by [52]:


$$\begin{array}{c} \begin{array}{c} \Phi_{k}^{m}(L)\nabla^{d_{k}}z_{k,t}=\mu_{k}+\beta_{k}^{\prime }{𝐙}_{t-1}^{\text{exo}}+\Theta_{k}^{m}(L)\epsilon_{k,t}, \end{array} \end{array}$$

where $\nabla^{d_{k}}=(1-L{)}^{d_{k}}$ is the differencing operator of order *d*_*k*_; $\Phi_{k}^{m}(L)$ and $\Theta_{k}^{m}(L)$ are the lag polynomials for the autoregressive (AR) and moving average (MA) components, respectively; $\mu_{k}$ is the intercept; ${𝐙}_{t-1}^{\text{exo}}$ is a vector of exogenous variables with coefficient vector $\beta_{k}^{\prime }$, and $\epsilon_{k,t}$ is the residual term. This provides the one-step-ahead forecast, ${\hat{z}}_{k,t+1|t}$.

To quantify the time-varying forecast uncertainty, we model the conditional variance of the ARIMAX residuals using a GARCH ($p_{k}^{v},q_{k}^{v}$) process:


$$\begin{array}{c} \begin{array}{c} h_{k,t+1}=w_{k}+\sum_{i=1}^{q_{k}^{v}}\alpha_{i,k}\varepsilon_{k,t+1-i}^{2}+\sum_{j=1}^{p_{k}^{v}}\beta_{j,k}h_{k,t+1-j},\;{\hat{\sigma }}_{k,t+1|t}=\sqrt{{\hat{h}}_{k,t+1|t}}, \end{array} \end{array}$$

where $h_{k,t}={𝔼}_{t-1}\left[\varepsilon_{k,t}^{2}\right]$ is the conditional variance and ${\hat{h}}_{k,t+1|t}$ is its one-step-ahead forecast implied by the recursion. The pair $\left({\hat{z}}_{k,t+1|t},{\hat{\sigma }}_{k,t+1|t}\right)$ constitutes the forward-looking signals passed to the next stage.

Predictors differ in persistence, seasonality, and noise, so bespoke models might lift fit for a few series. Our aim, however, is not to maximize first-stage fit but to produce comparable reliability scores in a consistent and fair way. Using a unified ARIMAX–GARCH modeling framework across all series ensures that cross-predictor differences in out-of-sample *R*^2^ reflect signal quality rather than model flexibility or researcher discretion.

### 3.2 Feature construction and dimension reduction

Stage 2 maps the predictor information available at time *t* into a feature vector **X**_*t*_ and uses it to forecast the next period equity premium *r*_*t*+1_. The key input to this stage is the collection of predictors described in Section 4.1, together with the forward-looking quantities generated in Stage 1. In what follows, we define the candidate feature pools and the optional processing steps—screening and dimension reduction—that we use to obtain parsimonious inputs for learning.

We begin with two base feature pools. The *Past* pool uses lagged predictor information only and is given by


$$\begin{array}{c} \begin{array}{c} {𝐅}_{t}^{p}={\left[z_{1,t},\cdots ,z_{K,t}\right]}^{⊤}. \end{array} \end{array}$$

The *Combined* pool augments each lagged predictor with a forward-looking pair produced in Stage 1. Specifically, for each predictor *k*, Stage 1 provides a one-step-ahead conditional mean forecast ${\hat{z}}_{k,t+1|t}$ and a one-step-ahead conditional residual volatility proxy ${\hat{\sigma }}_{k,t+1|t}$. We collect these into a predictor-level feature triplet


$$\begin{array}{c} \begin{array}{c} f_{k,t}=\left[z_{k,t},{\hat{z}}_{k,t+1|t},{\hat{\sigma }}_{k,t+1|t}\right], \end{array} \end{array}$$

and stack them across predictors to form


$$\begin{array}{c} \begin{array}{c} {𝐅}_{t}^{c}={\left[f_{1,t}^{⊤},\cdots ,f_{K,t}^{⊤}\right]}^{⊤}. \end{array} \end{array}$$

The Combined pool is designed to encode not only the current level of each predictor but also its anticipated movement and the uncertainty around that movement, allowing the model to exploit forward-looking information without introducing additional ad hoc state variables.

Economically, this augmentation is motivated by the idea that the current level of a predictor need not fully summarize the state relevant for next-period equity premia. Two periods with similar *z*_*k*,*t*_ can imply different required returns if the same predictor is expected to deteriorate further in one case but to stabilize or reverse in the other. The conditional mean forecast ${\hat{z}}_{k,t+1|t}$ therefore provides a parsimonious summary of the near-term direction in which the macro-financial state is likely to move, while ${\hat{\sigma }}_{k,t+1|t}$ captures how precisely that transition can be read at the forecast origin. In this sense, the uncertainty term is useful not only as a reliability measure for the generated mean forecast, but also as an indicator of local fragility or regime instability, under which the mapping from predictors to expected returns may differ. The Combined pool is thus intended to capture both where the economy currently is and where it is likely headed. This economic interpretation also motivates the admission rule introduced below: when the first-stage forward-looking signals are weakly forecastable, adding them is more likely to inject noise than to sharpen the return forecast.

Because the forward-looking components $\left({\hat{z}}_{k,t+1|t},{\hat{\sigma }}_{k,t+1|t}\right)$ are generated forecasts, their quality can vary substantially across predictors. To prevent noisy first stage forecasts from diluting the signal, we impose a predictor-level admission threshold τ when forming the Combined pool. Let $R_{OS,k}^{2}$ denote the out-of-sample predictive fit of the Stage 1 model for predictor *k*, computed using the benchmark definitions in Section 3.3. If $R_{OS,k}^{2}<\tau$, we drop only the forward-looking pair $\left({\hat{z}}_{k,t+1|t},{\hat{\sigma }}_{k,t+1|t}\right)$ from predictor *k* while always retaining the lagged level *z*_*k*,*t*_. This rule keeps the baseline information set intact and uses Stage 1 only when it delivers sufficiently reliable forward-looking content. The resulting post-threshold working pool therefore consists of all lagged predictor-levels together with only the admitted forward-looking columns.

To manage redundancy and improve parsimony, we optionally apply screening and/or dimension reduction to the base pools. First, we consider SHAP-based screening: we fit a preliminary model on the training data, compute feature attributions via TreeSHAP, and rank predictors by mean absolute SHAP importance. Implementation details are provided in Section 4.2.2. We then retain only the top *N* predictors and carry forward the corresponding features from the chosen base pool. Let ${𝐅}_{t}^{*}$ denote the resulting SHAP-screened feature vector.

Second, we apply linear dimension reduction to the working pool using either PCA or PLS. Let **F** denote the feature matrix over the training window formed from the chosen working pool (either directly from ${𝐅}_{t}^{p}$ or ${𝐅}_{t}^{c}$, or from the SHAP-screened ${𝐅}_{t}^{*}$), and let **r** denote the corresponding vector of target equity premia. PCA constructs orthogonal components that maximize the variance of the projected features by selecting loading vectors ${\left\{\omega_{k}\right\}}_{k=1}^{m}$ that solve


$$\begin{array}{c} \begin{array}{c} \omega_{k}=\arg\max_{\omega }\text{Var}(𝐅\omega )\;\text{s.t.}\;\;\omega^{\prime }\omega =1,\;\;\text{Cov}(𝐅\omega ,𝐅\omega_{j})=0\;\;\text{for}\;\;j<k, \end{array} \end{array}$$

PLS instead targets predictive directions by maximizing squared covariance with the equity premium, choosing ${\left\{\omega_{k}\right\}}_{k=1}^{m}$ according to


$$\begin{array}{c} \begin{array}{c} \omega_{k}=\arg\max_{\omega }{\text{Cov}}^{2}(𝐫,𝐅\omega )\;\text{s.t.}\;\;\omega^{\prime }\omega =1,\;\;\text{Cov}(𝐅\omega ,𝐅\omega_{j})=0\;\;\text{for}\;\;j<k, \end{array} \end{array}$$

In both cases, the constraints normalize the loadings and ensure that the extracted score vectors $𝐅\omega_{k}$ are mutually uncorrelated across components. Let ${\tilde{𝐅}}_{t}$ denote the resulting *m*-dimensional representation at time *t*, constructed by projecting the feature vector from the working pool on*t*o the selected directions.

Overall, depending on the specification under study, the Stage 2 input is taken from


$$\begin{array}{c} \begin{array}{c} {𝐗}_{t}\in \left\{{𝐅}_{t}^{p},{𝐅}_{t}^{c},{\tilde{𝐅}}_{t}\right\}. \end{array} \end{array}$$

Given the input representation **X**_*t*_, Stage 2 then fits a forecasting function g(·) and produces the one-step-ahead equity premium forecast


$$\begin{array}{c} \begin{array}{c} {\hat{r}}_{t+1}=g({𝐗}_{t}). \end{array} \end{array}$$

### 3.3 Performance evaluation

Our primary measure of forecast accuracy is the out-of-sample *R*^2^ ($R_{OS}^{2}$) in the sense of [3]. Let *r*_*t*+1_ denote the target at time *t* + 1 and ${\hat{r}}_{t+1}$ the corresponding one-step-ahead forecast formed using information available at time *t*. We define


$$\begin{array}{c} \begin{array}{c} R_{OS}^{2}=1-\frac{\sum_{t\in 𝒯}{\left(r_{t+1}-{\hat{r}}_{t+1}\right)}^{2}}{\sum_{t\in 𝒯}{\left(r_{t+1}-{\bar{r}}_{t+1}\right)}^{2}} \end{array} \end{array}$$

where ${\bar{r}}_{t+1}$ is the historical average return estimated through period *t*, and 𝒯 denotes the out-of-sample *t*est set disjoint from training and validation. Positive values indicate that the predictive model reduces mean squared error relative to the historical mean benchmark.

In addition, when evaluating one-step-ahead forecasts for highly persistent series as in our Stage 1 predictor forecasting, it is often more conservative to benchmark against a random-walk forecast. In that case, the out-of-sample statistic is computed as


$$\begin{array}{c} \begin{array}{c} R_{OS}^{2}=1-\frac{\sum_{t\in 𝒯}{\left(r_{t+1}-{\hat{r}}_{t+1}\right)}^{2}}{\sum_{t\in 𝒯}{\left(r_{t+1}-r_{t}\right)}^{2}}, \end{array} \end{array}$$

so that performance is assessed relative to the no change prediction *r*_*t*+1_ = *r*_*t*_. Throughout this paper, we use the historical mean benchmark for equity premium forecasting and for return or growth type targets, and the random walk benchmark for most persistent predictor-level targets in Stage 1. An exception is monthly inflation, for which predictor-level out-of-sample reliability is evaluated relative to a trailing 12-month average formed recursively from the most recent available observations and lagged by one period to preserve real-time timing. We adopt this benchmark because monthly inflation is persistent but noisy at short horizons, so a recent-year average provides a more meaningful and less noise-sensitive real-time baseline than a one-month no-change forecast.

Additionally, we also report the relative root mean squared error (RRMSE) as a supplementary scale-based measure of forecast accuracy. Let ${\tilde{r}}_{t+1}$ denote the relevant benchmark forecast. We define


$$\begin{array}{c} \begin{array}{c} RRMSE=\frac{\sqrt{\sum_{t\in 𝒯}{\left(r_{t+1}-{\hat{r}}_{t+1}\right)}^{2}}}{\sqrt{\sum_{t\in 𝒯}{\left(r_{t+1}-{\tilde{r}}_{t+1}\right)}^{2}}}. \end{array} \end{array}$$

Values below one indicate that the model outperforms the benchmark in root mean squared forecast error, while values above one indicate worse forecast accuracy. The benchmark used in RRMSE follows the same rule as for $R_{OS}^{2}$: The historical mean for equity premium forecasting and return or growth-type targets, and the random-walk forecast for highly persistent predictor-level targets.

To connect accuracy to market states, we also compute tail-conditional out-of-sample *R*^2^. For a quantile level q∈(0,1/2], let


$$\begin{array}{c} \begin{array}{c} \tau_{q}^{-}={Quantile}_{q}\left\{r_{s+1}:s\in 𝒯\right\},\;\tau_{q}^{+}={Quantile}_{1-q}\left\{r_{s+1}:s\in 𝒯\right\}, \end{array} \end{array}$$

and define the downside and upside index sets


$$\begin{array}{c} \begin{array}{c} {𝒟}_{q}\equiv \left\{t\in 𝒯:r_{t+1}\leq \tau_{q}^{-}\right\},\;{𝒰}_{q}\equiv \left\{t\in 𝒯:r_{t+1}\geq \tau_{q}^{+}\right\}. \end{array} \end{array}$$

The corresponding conditional *R*^2^ statistics are


$$\begin{array}{c} \begin{array}{c} R_{DOS}^{2}(q)=1-\frac{\sum_{t\in {𝒟}_{q}}(r_{t+1}-{\hat{r}}_{t+1}{)}^{2}}{\sum_{t\in {𝒟}_{q}}(r_{t+1}-{\bar{r}}_{t+1}{)}^{2}},\;R_{UOS}^{2}(q)=1-\frac{\sum_{t\in {𝒰}_{q}}(r_{t+1}-{\hat{r}}_{t+1}{)}^{2}}{\sum_{t\in {𝒰}_{q}}(r_{t+1}-{\bar{r}}_{t+1}{)}^{2}}. \end{array} \end{array}$$

Both metrics evaluate the reduction in squared forecast errors within either left-tail (downside) or right-tail (upside) months of the test period, relative to the same benchmark as above. Economically, a positive $R_{DOS}^{2}(q)$ indicates improved accuracy precisely in adverse market states–relevant for downside risk management–whereas a positive $R_{UOS}^{2}(q)$ reflects better accuracy in favorable states and stronger upside participation.

## 4 Data and experimental setup

We study monthly U.S. equity premium predictability using the two stage design introduced earlier. Here we detail the practical setup: data, preprocessing, feature construction options, and the training and evaluation framework.

### 4.1 Data

Our sample spans January 1952 to December 2024. The equity premium is measured as the monthly market return minus the risk-free rate. Primary results are reported for the S&P 500, with robustness checks on the CRSP value-weighted index. Our baseline follows the 17 predictors of [1], augmented with five variables highlighted as promising in more recent work [2]: a composite index of technical indicators (tchi), tail risk (tail), average stock correlation (avgcor), the output gap (ogap), and growth in personal consumption expenditures (gpce). A key inclusion criterion is the availability of a continuous series back to at least 1952, ensuring a sufficiently long sample for robust out-of-sample evaluation.

To support economic interpretation and to stabilize later attribution exercises, we organize the predictors into six families following [1,2]—Valuation; Rates, Term Structure & Credit; Macroeconomic; Equity Issuance & Financing; Market Risk & Comovement; and Technical. This classification is also the one used later when we aggregate importance measures at the group-level.

Because our two stage design constructs forward-looking signals at the predictor-level, all subsequent steps rely on a real-time predictor panel that respects publication lags and appropriately aligns mixed frequencies. We describe these preprocessing and alignment rules—together with the Stage 1 forecasting procedure built on top of them—in Section 4.2.1.

### 4.2 Implementation details

This section describes the empirical implementation of our framework in a real-time setting. Building on the data described in Section 4.1, we summarize the preprocessing required to avoid look-ahead, the construction of the feature sets used in the forecasting experiments, and the training and evaluation protocol adopted throughout.

#### 4.2.1 Stage 1: Preprocessing and forecasting each predictor.

##### Data preprocessing and mixed-frequency alignment

Stage 1 is implemented on a real-time predictor panel that respects publication lags and aligns mixed frequency series without look-ahead. We first shift each predictor by a fixed release lag based on its native frequency—one month for monthly series, three months for quarterly series, and one year for annual series—before any further transformation or standardization. Because each predictor is forecast in turn using the remaining variables as candidate exogenous regressors, frequency alignment is performed relative to the target variable in each forecasting model: regressors observed at the same frequency enter under the same lagged timing; when the target is higher frequency, lower frequency regressors are carried forward using their most recently released observation until the next release; and when the target is lower frequency (e.g., quarterly or annual), higher frequency regressors are aggregated to the target periodicity as described below.

Concretely, the aggregation step follows standard stock-flow conventions so that the mapped regressor preserves the variable’s economic interpretation. In particular, variables naturally interpreted as end of period state quantities—such as interest rate levels and spreads (e.g., lty, tbl, tms, dfy) and valuation ratios (e.g., d/p, d/y, e/p, d/e, b/m), as well as indices such as tchi and ogap—are mapped to the period-end value. Variables representing within-period conditions such as average states or risk—such as inflation and market risk/comovement measures (e.g., infl, svar, avgcor, tail)—are mapped to the period average. Cumulative flow series are mapped to the period sum (e.g., ntis), and return type series are mapped to period returns via geometric compounding (e.g., dfr, ltr).

To limit obvious redundancy in Stage 1, we did not impose a blanket exclusion rule at the level of the six broad predictor families in Table 1. Instead, the candidate exogenous set for each target predictor was restricted only in a limited, pair-specific way, excluding variables that were regarded as mechanically overlapping or as near-equivalent representations of the same underlying quantity. This mainly applied to closely related measures such as alternative valuation ratios or tightly linked rate/spread variables. Accordingly, the family classification in Table 1 should be interpreted as descriptive and used for economic organization, rather than as the formal criterion determining admissible exogenous regressors in Stage 1. The empirical implementation uses a single admissible exogenous regressor for each target predictor; Table 2 reports the selected regressor for each series.

**Table 1:** Predictor families, variable names, and native frequencies.

Category	Variable Name	Frequency
Valuation	Dividend-Price Ratio (d/p)	Monthly
	Dividend Yield (d/y)	Monthly
	Earnings-Price Ratio (e/p)	Monthly
	Dividend Payout Ratio (d/e)	Monthly
	Book-to-Market Ratio (b/m)	Monthly
Rates, Term Structure & Credit	Treasury-Bill Rate (tbl)	Monthly
	Long-Term Yield (lty)	Monthly
	Long-Term Return (ltr)	Monthly
	Term Spread (tms)	Monthly
	Default Yield Spread (dfy)	Monthly
	Default Return Spread (dfr)	Monthly
Macroeconomic	Inflation (infl)	Monthly
	Investment-to-Capital Ratio (i/k)	Quarterly
	Consumption–Wealth–Income Ratio (cay)	Quarterly
	Output Gap (ogap)	Quarterly
	Personal Consumption Growth (gpce)	Yearly
Equity Issuance & Financing	Net Equity Issuance (ntis)	Monthly
	Percent Equity Issuing (eqis)	Yearly
Market Risk & Comovement	Stock Market Variance (svar)	Monthly
	Average Correlation (avgcor)	Monthly
	Tail Risk (tail)	Monthly
Technical	Composite Technical Index (tchi)	Monthly

**Table 2:** ARIMAX specifications and in-/out-of-sample *R*^2^ for each target variable. For each series we report the selected exogenous predictor, the optimal ARIMAX order (*p*^*m*^, *d*, *q*^*m*^) – where *p*^*m*^ denotes the number of autoregressive lags, *d* the order of differencing, and *q*^*m*^ the number of moving-average lags – and the resulting in-sample ($R_{IS}^{2}$) and out-of-sample ($R_{OS}^{2}$) values.

Variable	Exo. vars	Order	$R_{IS}^{2}$	$R_{OS}^{2}$
d/e	gpce	(2,0,1)	0.4525	0.3750
i/k	ogap	(1,0,0)	0.3738	0.3125
svar	avgcor	(0,0,2)	0.3044	0.2415
d/y	tchi	(1,1,0)	0.2236	0.1906
avgcor	dfy	(1,0,2)	0.2426	0.1896
dfy	avgcor	(1,0,1)	0.1616	0.1440
tail	d/y	(2,0,2)	0.0990	0.1145
tbl	tchi	(2,0,0)	0.1271	0.1143
tchi	ogap	(1,0,1)	0.1446	0.1017
infl	e/p	(2,0,2)	0.3221	0.0882
ogap	dfy	(1,0,1)	0.1471	0.0674
e/p	tbl	(0,1,0)	0.0488	0.0361
cay	gpce	(1,0,0)	0.0866	0.0343
eqis	ogap	(1,0,0)	0.1853	0.0334
tms	dfy	(2,0,0)	0.0559	0.0257
d/p	eqis	(0,1,0)	0.0296	0.0248
lty	infl	(1,0,0)	0.0152	0.0110
ntis	b/m	(1,0,0)	0.0220	−0.0010
b/m	lty	(1,0,0)	0.0227	−0.0011
dfr	e/p	(1,0,1)	0.0139	−0.0048
ltr	tms	(2,0,2)	0.0177	−0.0098
gpce	ogap	(0,0,0)	0.0276	−0.0241

##### Forecasting model and expanding window refits

The objective of Stage 1 is to construct, for each predictor *z*_*k*_, a forward-looking pair $\left({\hat{z}}_{k,t+1|t},{\hat{\sigma }}_{k,t+1|t}\right)$ consisting of the one-step-ahead conditional mean and the conditional residual volatility for its forecast uncertainty. We implement this by estimating an ARIMAX model for the conditional mean and a GARCH model for the residual variance on the real-time dataset described above, maintaining the exclusion map throughout. The procedure is run on an expanding window with annual refits. We reserve January 1952 through December 1971 as an initial training period and then produce the first one-step-ahead forecasts for 1972, repredicting the models each year using the expanding sample available at that point.

To stabilize the mean component, the ARIMAX orders (*p*^*m*^, *d*, *q*^*m*^) are selected once at the initial calibration using the Akaike information criterion (AIC) from a compact grid—*p*^*m*^ and *q*^*m*^ ranging from 0 to 2, and *d* equal to 0 or 1—and then held fixed. In contrast, the GARCH orders (*p*^*v*^, *q*^*v*^) are reselected via AIC at each annual refit, allowing the volatility dynamics to adapt over time; for this, the GARCH order *p*^*v*^ is set to 1 or 2, and the ARCH order *q*^*v*^ ranges from 0 to 2. This unified template is intentionally parsimonious: our aim is not to maximize first stage fit for any single predictor, but to generate comparable one-step-ahead forecasts across series so that cross-predictor differences primarily reflect signal quality rather than model flexibility.

From the resulting expanding window forecasts, we compute a predictor-level out-of-sample $R_{OS}^{2}$ as a reliability score, using the benchmark definitions in Section 3.3. These scores are subsequently used to screen the forward-looking components when constructing the Combined feature set.

#### 4.2.2 Stage 2: Feature construction, reduction, and learning.

Using the Stage 1 outputs, we construct the feature sets used for equity premium forecasting. We consider two base pools: the Past set, which uses lagged predictors only, and the Combined set, which augments each lag with the Stage 1 forward-looking pair, as defined in Section 3.2. When forming the Combined pool, we apply a predictor-level admission threshold τ∈{0.0,0.05,0.1,0.15,0.2} to each predictor’s Stage 1 reliability score $R_{OS}^{2}$. The lower bound τ=0 should be interpreted as the weakest admissible screen, excluding only predictors with negative Stage 1 out-of-sample *R*^2^, whereas the positive cutoffs form a fixed coarse sensitivity grid that imposes progressively stricter admission rather than an optimized tuning rule. If predictor *k*’s reliability falls below τ, we drop only its forward-looking pair while always retaining the lagged predictor-level in the feature pool.

After constructing the working pool, we standardize the resulting features, with one exception: we keep the conditional volatility forecasts on their raw scale to preserve the informational content of their magnitude. We then consider additional steps to address multicollinearity and to obtain parsimonious representations. First, we optionally apply SHAP-based screening. We fit a preliminary tree-based model on the training window, compute TreeSHAP attributions for observations in the corresponding validation block, and rank predictors by their mean absolute SHAP values averaged over the validation block. We form predictor-level importance scores by aggregating the mean absolute SHAP values over the validation block and retain the top *N* predictors, with N∈{5,10,15,20}, carrying forward the corresponding features to the working pool. Second, we perform PCA or PLS either on the unscreened pool or on the SHAP-screened pool; for completeness, we also report PCA/PLS applied directly to the full feature set without SHAP screening.

Models are retrained annually on an expanding training window with a fixed-length rolling validation block for hyperparameter selection. In our baseline implementation, we use random forests to implement the forecasting rule due to their ability to capture nonlinearities and interactions while mitigating overfitting in high-dimensional settings. For the S&P 500, the initial training window is 1972–1991 and the moving validation block is 1992–1999; out-of-sample evaluation runs 2000–2024. To assess robustness across equity universes, we replicate the same evaluation protocol on the CRSP value-weighted index using the identical training, validation, and test windows; detailed results are reported in S2 Appendix. Unless otherwise noted, the validation criterion is mean squared error. Random forests use 300 trees with maximum depth fixed at 1. We tune the fraction of features considered at each split over {0.02,0.04} and the minimum fraction of samples per leaf over {0.001,0.002,0.004,0.008,0.01,0.02,0.03,0.05,0.1}. For PCA/PLS, the number of components is selected from {1,2,3,5,8,15}. Tail-conditional accuracy measures are computed in the left- and right-tail months of the test window; main results are reported at q = 10%, and a sensitivity analysis over additional quantile levels is provided in S3 Appendix. As a further robustness check on the choice of learner, we repeat the Stage 2 exercise using gradient-boosted tree ensembles—specifically XGBoost and LightGBM—in place of random forests; these results are also reported in S2 Appendix.

## 5 Results and discussion

This section reports the empirical results and discusses their implications for real-time equity premium forecasting, with an emphasis on how selective admission of forward-looking signals affects both statistical accuracy and portfolio performance.

### 5.1 Stage 1 forecastability and reliability screening

Before turning to equity premium forecasting results, we first summarize how forecastable the underlying macro–financial predictors are in real time, since this stage determines which forward-looking signals are admitted into the combined feature pool. In Stage 1, we fit a parsimonious ARIMAX specification with a single admissible exogenous regressor for each predictor *z*_*k*_ to generate one-step-ahead conditional mean forecasts ${\hat{z}}_{k,t+1|t}$, and pair them with a one-step-ahead residual uncertainty proxy ${\hat{\sigma }}_{k,t+1|t}$; for each target series, we identify the single best-performing exogenous regressor through the cross-prediction procedure described in Section 4.2.1.

Table 2 reports, for each predictor, the selected exogenous variable, the ARIMAX order (*p*^*m*^, *d*, *q*^*m*^) determined by the AIC, and the resulting in-sample and out-of-sample fit statistics, where out-of-sample performance is evaluated using the benchmark rules in Section 3.3. Supplementary assessment of the baseline Stage 1 ARIMAX–GARCH specification is reported in S1 Appendix.

A clear dispersion emerges across predictors. Several series—such as investment i/k, payout d/e, and market-wide risk/comovement measures including svar and avgcor—exhibit materially positive out-of-sample performance in this first stage exercise, whereas selected valuation, issuance, and macroeconomic variables such as b/m, ntis, and gpce display little to no incremental predictive content, with $R_{OS}^{2}$ near zero or negative; rate, term-structure, and credit variables show mixed results.

We carry these predictor-level $R_{OS}^{2}$ values forward as reliability scores that discipline the construction of the combined feature set in Stage 2. Concretely, when forming the combined pool, we apply the threshold τ to each predictor’s Stage 1 reliability and drop only its forward-looking pair when the reliability falls below τ, while always retaining the contemporaneous lag *z*_*k*,*t*_. This design makes the role of forward-looking information transparent: it is included only when the underlying predictor demonstrates adequate real-time forecastability, and it allows the subsequent empirical analysis to isolate the incremental value of admitting these generated signals relative to using lagged predictors alone.

### 5.2 Main Results and Portfolio Performance

This section reports our main Stage 2 equity premium forecasting results and evaluates their economic value. We summarize statistical accuracy using out-of-sample *R*^2^ measures and translate each one-step-ahead forecast into a constrained mean-variance portfolio to assess realized performance.

Following [3], at each time *t*, the portfolio weight allocated to the risky asset is determined by:


$$\begin{array}{c} \begin{array}{c} w_{p,t}=\min\left({\bar{w}}_{p},\max\left({\underset{―}{w}}_{p},\frac{{\hat{r}}_{t+1}}{\gamma {\hat{\sigma }}_{p,t+1}^{2}}\right)\right), \end{array} \end{array}$$

where ${\hat{\sigma }}_{p,t+1}^{2}$ is a backward-looking variance estimate computed from a rolling 5-year window, γ=3 is the risk aversion coefficient, and ${\bar{w}}_{p}=1.5$ and ${\underset{―}{w}}_{p}=0.0$ impose no short selling and leverage constraints. Robustness to alternative volatility estimators used in this portfolio-scaling rule is reported in S4 Appendix. Portfolio performance is reported on an annualized basis. Let $\mu_{p}$ and $\sigma_{p}$ denote the annualized mean excess return and volatility of the portfolio. We report the Sharpe ratio $\mu_{p}/\sigma_{p}$, and the certainty equivalent return


$$\begin{array}{c} \begin{array}{c} \text{CER}=\mu_{p}-\frac{1}{2}\gamma \sigma_{p}^{2}. \end{array} \end{array}$$

We also report maximum drawdown


$$\begin{array}{c} \begin{array}{c} \text{MDD}=\max_{t}\left(1-\frac{W_{t}}{\max_{0\leq s\leq t}W_{s}}\right), \end{array} \end{array}$$

where *W*_*t*_ is the cumulative wealth. To emphasize downside risk, we compute the Sortino ratio $\mu_{p}/\sigma_{\text{down}}$ with


$$\begin{array}{c} \begin{array}{c} \sigma_{\text{down}}=\sqrt{𝔼\left[\min(r_{p},0{)}^{2}\right]}, \end{array} \end{array}$$

and we quantify trading intensity by annualized turnover


$$\begin{array}{c} \begin{array}{c} \text{Turnover}=12\times \frac{1}{T}\sum_{t=1}^{T}\left\|w_{p,t}-w_{p,t-1}\right\|. \end{array} \end{array}$$

Throughout Tables 3–5, “Past” denotes models that use lagged predictors only, whereas “Comb.” denotes the combined feature set that augments these lags with Stage 1 forward-looking signals admitted under the predictor-level reliability threshold τ. “SHAP-PCA/PLS” indicates that predictors are first pre-screened by SHAP importance and then reduced by PCA or PLS before forecasting; τ is the minimum individual Stage 1 out-of-sample *R*^2^ required for a predictor’s forward-looking forecast to enter the combined set. We first assess the economic value of the forecasts via portfolio outcomes and then examine statistical predictive accuracy.

**Table 3:** Portfolio performance by feature representation and estimation method. The table reports Sharpe ratio, Sortino ratio, certainty equivalent return (CER) with risk aversion γ=3, maximum drawdown (MDD), and turnover for various forecasting strategies. The reliability threshold τ refers to the minimum individual predictor out-of-sample *R*^2^ from Stage 1 required for its forward-looking forecast to be included in the combined feature set.

Method	Feature	τ	Sharpe	Sortino	CER	MDD	Turnover
Buy & Hold	Past	–	0.4560	0.6626	0.0347	0.5022	–
CAPM			0.2921	0.3949	0.0109	0.5127	2.7710
FF3			0.2600	0.3606	0.0025	0.5936	5.3486
–	Past	–	0.2719	0.3599	0.0048	0.5909	0.8173
	Comb.	0.00	0.1796	0.2328	−0.0082	0.5545	0.7960
		0.05	0.2800	0.3721	0.0062	0.5545	0.8026
		0.10	0.3507	0.4788	0.0171	0.5469	0.7645
		0.15	0.2915	0.3906	0.0070	0.5880	0.9704
		0.20	0.2624	0.3471	0.0035	0.5493	0.9297
		PCA	Past	–	0.3176	0.4436	0.0107	0.6645	0.6818
Comb.	0.00		0.3835	0.5303	0.0227	0.5556	1.0121
	0.05		0.3345	0.4696	0.0131	0.6630	0.7619
	0.10		0.3224	0.4515	0.0111	0.6641	0.6648
	0.15		0.2451	0.3264	−0.0000	0.6645	0.7151
	0.20		0.2923	0.3945	0.0080	0.6645	0.5879
PLS	Past	–	0.5117	0.7303	0.0436	0.6561	1.7927
	Comb.	0.00	0.2710	0.3625	0.0049	0.5397	1.6234
		0.05	0.4436	0.6208	0.0328	0.4673	2.6328
		0.10	0.4937	0.6929	0.0403	0.5457	2.5089
		0.15	0.5510	0.8294	0.0466	0.4595	2.8478
		0.20	0.3515	0.4913	0.0193	0.3994	1.5598
SHAP-PCA	Past	–	0.4323	0.5921	0.0307	0.5409	1.4063
	Comb.	0.00	0.4152	0.5950	0.0275	0.4856	1.5568
		0.05	0.5006	0.7350	0.0414	0.3453	1.5452
		0.10	0.4773	0.6993	0.0377	0.4602	1.8563
		0.15	0.3767	0.5287	0.0188	0.6645	0.7999
		0.20	0.3399	0.4666	0.0157	0.6509	0.8672
SHAP-PLS	Past	–	0.5544	0.8163	0.0502	0.3897	2.0991
	Comb.	0.00	0.4495	0.6705	0.0337	0.3591	1.7891
		0.05	0.6390	0.9958	0.0619	0.3643	2.4373
		0.10	0.5924	0.9129	0.0578	0.4449	1.8675
		0.15	0.3208	0.4379	0.0132	0.5273	1.5556
		0.20	0.6835	1.1139	0.0653	0.3254	2.5964

**Table 4:** Net of transaction cost portfolio performance by feature representation and estimation method. All entries are computed after deducting proportional transaction costs of 25 basis points per unit of turnover. The table reports Sharpe ratio, Sortino ratio, certainty equivalent return (CER) with risk aversion γ=3, maximum drawdown (MDD), and turnover for various forecasting strategies. The combined feature set admits forward-looking signals only for predictors whose Stage 1 out-of-sample *R*^2^ exceeds the reliability threshold τ=0.10.

Method	Feature	Sharpe	Sortino	CER	MDD	Turnover
Buy & Hold	Past	0.4560	0.6626	0.0347	0.5022	–
CAPM		0.2446	0.3276	0.0041	0.5167	2.7710
FF3		0.1783	0.2441	−0.0109	0.5964	5.3486
–	Past	0.2593	0.3426	0.0028	0.5920	0.8173
	Comb.	0.3392	0.4622	0.0152	0.5471	0.7645
PCA	Past	0.3077	0.4292	0.0091	0.6645	0.6818
	Comb.	0.3127	0.4373	0.0095	0.6642	0.6648
PLS	Past	0.4861	0.6915	0.0393	0.6564	1.7927
	Comb.	0.4525	0.6316	0.0341	0.5492	2.5089
SHAP-PCA	Past	0.4103	0.5603	0.0272	0.5414	1.4063
	Comb.	0.4498	0.6565	0.0329	0.4695	1.8563
SHAP-PLS	Past	0.5214	0.7631	0.0450	0.4016	2.0991
	Comb.	0.5653	0.8679	0.0530	0.4612	1.8675

**Table 5:** Out-of-sample *R*^2^ for the equity risk premium forecasts by feature representation, estimation method, and predictor level threshold τ. The table reports the in-sample *R*^2^ and three out-of-sample *R*^2^ measures relative to the historical mean benchmark. The threshold τ refers to the minimum individual predictor out-of-sample *R*^2^ from Stage 1 required for its forward-looking forecast to be included in the combined feature set. $R_{DOS}^{2}$ and $R_{UOS}^{2}$ denote out-of-sample *R*^2^ in downside (left-tail) and upside (right-tail) months, as defined in Section 3.3.

Method	Feature	τ	$R_{IS}^{2}$	$R_{OS}^{2}$	$R_{DOS}^{2}$	$R_{UOS}^{2}$	RRMSE
–	Past	–	0.0239	−0.0078	−0.0218	−0.0017	1.0039
	Comb.	0.00	0.0267	−0.0153	−0.0149	−0.0227	1.0076
		0.05	0.0254	−0.0075	−0.0192	−0.0008	1.0037
		0.10	0.0240	−0.0006	−0.0184	0.0173	1.0003
		0.15	0.0262	−0.0063	−0.0323	0.0190	1.0032
		0.20	0.0255	−0.0079	−0.0199	0.0015	1.0039
PCA	Past	–	0.0231	−0.0015	−0.0160	0.0241	1.0008
	Comb.	0.00	0.0225	0.0015	−0.0186	0.0213	0.9993
		0.05	0.0227	0.0024	−0.0263	0.0374	0.9988
		0.10	0.0214	0.0020	−0.0208	0.0314	0.9990
		0.15	0.0231	−0.0052	−0.0190	0.0095	1.0026
		0.20	0.0227	0.0004	−0.0047	0.0093	0.9998
PLS	Past	–	0.0523	0.0021	−0.0115	0.0029	0.9989
	Comb.	0.00	0.0338	−0.0073	−0.0072	0.0055	1.0036
		0.05	0.0660	0.0062	0.0394	−0.0457	0.9969
		0.10	0.0653	0.0040	0.0386	−0.0525	0.9980
		0.15	0.0649	0.0039	0.0783	−0.0887	0.9980
		0.20	0.0320	−0.0040	0.0087	−0.0110	1.0020
SHAP-PCA	Past	–	0.0281	0.0041	−0.0002	−0.0033	0.9979
	Comb.	0.00	0.0263	0.0157	0.0062	0.0236	0.9921
		0.05	0.0297	0.0078	0.0127	0.0087	0.9961
		0.10	0.0240	0.0056	−0.0355	0.0293	0.9972
		0.15	0.0259	0.0068	−0.0377	0.0589	0.9966
		0.20	0.0313	0.0035	−0.0096	0.0063	0.9982
SHAP-PLS	Past	–	0.0414	0.0153	0.0072	0.0212	0.9923
	Comb.	0.00	0.0279	−0.0022	0.0276	−0.0241	1.0011
		0.05	0.0310	0.0163	0.0300	−0.0361	0.9918
		0.10	0.0463	0.0212	0.0063	0.0478	0.9893
		0.15	0.0253	−0.0054	0.0246	−0.0261	1.0027
		0.20	0.0453	0.0088	0.0734	−0.0593	0.9956

Table 3 provides a direct economic lens on the Stage 2 forecasts by comparing the implied mean-variance portfolios to standard benchmarks. A simple buy-and-hold allocation delivers a Sharpe ratio of 0.46 with an MDD of 0.50, while the conditional CAPM and FF3 benchmark strategies exhibit notably lower risk-adjusted performance and comparable or larger drawdowns.

Our primary focus is the incremental benefit of augmenting lagged predictors with the admitted forward-looking signals relative to using lagged predictors alone. The evidence in Table 3 suggests that such gains are possible, but not uniform across representations or threshold choices. In the raw feature block, a moderate positive threshold performs better than both the past-only baseline and the weakest screen: at τ=0.10, the combined specification raises the Sharpe ratio from 0.2719 to 0.3507 and lowers MDD from 0.5909 to 0.5469 relative to Past, although it still remains below buy-and-hold.

PCA offers a weaker investment profile overall: Combined variants are sometimes mildly better than Past in Sharpe or CER, but drawdowns remain high and the gains are not consistent across τ. By contrast, PLS-based representations benefit more from selective admission. The strongest gross PLS combined case occurs at τ=0.15, where Sharpe, Sortino, and CER all exceed the past-only PLS specification and MDD falls from 0.6561 to 0.4595.

SHAP screening sharpens this pattern. SHAP-PCA combined at τ=0.05 improves both Sharpe and downside protection relative to its Past counterpart, while SHAP-PLS delivers the strongest gross performance in the table. The τ=0.20 specification reaches a high performance overall, and the τ=0.05 and τ=0.10 variants also remain stronger than the past-only SHAP-PLS baseline on risk-adjusted return measures. Overall, Table 3 suggests that forward-looking augmentation can yield economically meaningful gains, but mainly in selected representations and thresholds rather than uniformly across all combined designs.

Table 4 complements this picture by evaluating net of transaction cost portfolio performance at the representative interior threshold τ=0.10 after deducting proportional transaction costs. The raw combined specification still improves on raw Past in Sharpe, CER, and MDD, whereas PCA remains essentially unchanged. For PLS, the combined design continues to reduce drawdown materially but no longer improves Sharpe or CER relative to PLS Past. SHAP-PCA combined remains competitive, improving on its Past counterpart in Sharpe, Sortino, CER, and MDD, and SHAP-PLS combined remains the strongest net of transaction cost combined specification, with a Sharpe ratio of 0.5653 and a CER of 0.0530, while also reducing turnover relative to past-only SHAP-PLS. S4 Appendix additionally reports the corresponding net of transaction cost results over the full τ-grid, as well as sensitivity checks under 10 and 50 basis point transaction cost assumptions.

From a practitioner’s perspective, these results suggest that the additional modeling complexity is justified only when it survives a joint screen based on risk-adjusted return, drawdown control, and trading intensity after costs. Under that lens, the most compelling specification in the main text is SHAP-PLS at τ=0.10, not because the two-stage framework uniformly dominates simpler strategies, but because this specification remains competitive after costs on the metrics practitioners are most likely to care about. By contrast, other combined designs appear more mixed: some improve drawdown without clearly improving Sharpe, while others show only modest net gains once turnover is taken into account. The practical value of the framework should therefore be viewed as selective rather than universal, with the strongest case arising when forward-looking signals are paired with disciplined admission and supervised screening.

A well-known intuition from [3] is that even small gains in out-of-sample *R*^2^ can deliver large economic value for a mean-variance investor. By contrast, [53] emphasize that minimizing a forecasting loss need not maximize a portfolio objective, so higher out-of-sample *R*^2^ need not translate into a higher Sharpe ratio. Our evidence reflects both sides of this wedge: some specifications that rank favorably by aggregate $R_{OS}^{2}$ do not deliver commensurate gains in realized portfolio performance, while some economically attractive variants show only modest aggregate improvements in statistical fit. With this distinction in mind, Table 5 summarizes the statistical forecasting evidence more directly.

Table 5 summarizes predictive performance in Stage 2 across feature families, dimension-reduction choices, and the predictor-level admission threshold τ. It reports in-sample *R*^2^ ($R_{IS}^{2}$), aggregate out-of-sample *R*^2^ relative to the historical mean benchmark ($R_{OS}^{2}$), the tail-conditional measures $R_{DOS}^{2}$ and $R_{UOS}^{2}$, and RRMSE. A first takeaway is that forward-looking signals are not *free*: under the weakest admissible screen (τ=0), several combined specifications remain weak or negative in aggregate out-of-sample fit. A moderate positive threshold can stabilize performance in some blocks, but not mechanically. In the non-SHAP PCA block, aggregate $R_{OS}^{2}$ moves from −0.0015 under Past to 0.0015 at τ=0 and 0.0024 at τ=0.05 under the combined feature set. In the PLS block, Past yields 0.0021, while the combined set improves to 0.0062, 0.0040, and 0.0039 at τ=0.05, 0.10, and 0.15, respectively, before turning negative again at τ=0.20. Thus τ should again be interpreted as a selectivity device that trades off signal quality against information retention, rather than as a monotone tuning parameter.

Second, SHAP screening yields the strongest and most consistent performance. SHAP-PCA Past already produces positive aggregate $R_{OS}^{2}$ (0.0041), and all of its combined variants remain positive, with the strongest aggregate fit at τ=0 (0.0157) and RRMSE as low as 0.9921. SHAP-PLS performs even better overall: The past-only version reaches 0.0153, while the combined set peaks at 0.0212 for τ=0.10 and 0.0163 for τ=0.05, with the τ=0.10 specification also delivering the lowest RRMSE in the table (0.9893). These patterns are consistent with the view that target-aligned SHAP screening removes weak or redundant inputs before the representation step.

Finally, the tail-conditional diagnostics show that predictive gains are state dependent. PCA and SHAP-PCA combined specifications tend to lean more toward upside accuracy, as reflected in positive $R_{UOS}^{2}$ alongside negative $R_{DOS}^{2}$ at several thresholds. By contrast, combined PLS specifications often load more heavily on downside months: for example, PLS with combined feature set at τ=0.15 yields $R_{DOS}^{2}=0.0783$ but $R_{UOS}^{2}=-0.0887$. SHAP-PLS is more mixed across thresholds, with τ=0.10 positive in both tails but τ=0.20 strongly skewed toward downside accuracy ($R_{DOS}^{2}=0.0734$, $R_{UOS}^{2}=-0.0593$). These state-conditional patterns help explain why aggregate forecast fit and portfolio outcomes need not move one-for-one, especially for path-dependent objects such as maximum drawdown.

### 5.3 Additional validation and robustness checks

#### 5.3.1 Forecast significance tests.

Table 6 complements the $R_{OS}^{2}$-based comparisons with formal forecast significance tests. We use the one-sided Diebold–Mariano (DM) test as our primary loss based test throughout. For comparisons against the historical mean benchmark, we additionally report the Clark–West (CW) statistic. The reason is that the historical mean is a parsimonious benchmark, whereas our competing specifications are richer conditional forecasting models estimated recursively from larger feature sets. In such settings, the larger model can be penalized in raw MSPE comparisons by estimation noise even when it contains incremental predictive information, which is precisely the case for which the CW adjustment was developed [54].

**Table 6:** Diebold–Mariano (DM) and Clark–West (CW) test statistics for one-step-ahead equity risk premium forecasts. The left block reports one-sided benchmark relative test statistics against the historical mean forecast. The Pairwise block reports one-sided DM test statistics comparing the Combined specification with the matched Past specification within the same method and threshold. $\Delta R_{OS}^{2}$ denotes the difference in out-of-sample *R*^2^ between the Combined and Past specifications. Superscripts *, **, and *** denote statistical significance at the 10%, 5%, and 1% levels, respectively, based on one-sided tests.

Method	Feature	τ	Benchmark		Pairwise	
DM	CW	$\Delta R_{OS}^{2}$	DM
–	Past	–	−1.6444	−1.4620	–	–
		0.00	−2.1952	−1.9953	−0.0075	−1.5641
		0.05	−1.3532	−1.1839	0.0003	0.1124
	Comb.	0.10	−0.1434	0.0571	0.0072	1.9232^**^
		0.15	−1.2297	−1.0243	0.0014	0.5359
		0.20	−1.5292	−1.3580	−0.0001	−0.0593
PCA	Past	–	−0.2603	0.1404	–	–
		0.00	0.2793	0.5190	0.0030	0.3750
		0.05	0.3971	0.6609	0.0039	1.1370
	Comb.	0.10	0.3573	0.6170	0.0035	1.2395
		0.15	−0.8946	−0.6719	−0.0036	−0.6204
		0.20	0.1110	0.3588	0.0019	0.4802
PLS	Past	–	0.1162	1.3286^*^	–	–
		0.00	−1.2492	−0.8488	−0.0094	−0.4594
		0.05	0.3705	1.4929^*^	0.0041	0.2243
	Comb.	0.10	0.2268	1.3195^*^	0.0019	0.0958
		0.15	0.2047	1.2996^*^	0.0018	0.0860
		0.20	−0.5746	−0.1686	−0.0061	−0.3068
SHAP-PCA	Past	–	0.5902	0.9350	–	–
		0.00	1.3612^*^	1.6773^**^	0.0116	0.9119
		0.05	1.2680	1.5542^*^	0.0037	0.4807
	Comb.	0.10	0.4406	1.0995	0.0015	0.0988
		0.15	0.8074	1.1501	0.0027	0.2471
		0.20	0.5909	1.0000	−0.0006	−0.0643
SHAP-PLS	Past	–	1.4629^*^	2.0006^**^	–	–
		0.00	−0.2080	0.3315	−0.0176	−1.2809
		0.05	0.9503	1.8074^**^	0.0010	0.0574
	Comb.	0.10	1.1704	2.1191^**^	0.0059	0.4779
		0.15	−0.4431	0.1126	−0.0207	−1.3178
		0.20	0.4253	1.6144^*^	−0.0066	−0.3513

Accordingly, the left block of Table 6 reports benchmark relative DM and CW test statistics, where larger positive values indicate stronger evidence that the reported specification improves upon the historical mean benchmark. The right block reports the paired comparison between the Combined and matched Past specifications within each method and threshold; there, $\Delta R_{OS}^{2}\;\equiv \;R_{OS}^{2}(Comb.)\;-R_{OS}^{2}(Past)$, and positive values of both $\Delta R_{OS}^{2}$ and the DM statistic favor the Combined specification.

The benchmark-relative results in the left block of Table 6 show that the DM evidence is selective rather than broad-based. The clearest joint support from both DM and CW appears in the SHAP-screened specifications, most notably for SHAP-PCA with τ=0 and for the SHAP-PLS Past specification. More generally, benchmark-relative support is visibly stronger under CW than under DM. This pattern is most apparent for the PLS and SHAP-PLS families, where several Combined specifications receive positive CW support even when the corresponding DM statistic does not reach conventional significance. We interpret this asymmetry cautiously: It suggests that some richer conditional specifications contain incremental information relative to the historical mean benchmark, while the raw loss-differential evidence remains modest.

The right block of Table 6 asks a stricter question: whether augmenting past predictors with admitted forward-looking signals improves upon the matched Past specification within the same representation. Here the evidence is more limited. The clearest result is the unreduced specification at τ=0.10, where the Combined design delivers a positive $\Delta R_{OS}^{2}$ together with a statistically significant DM statistic. PCA at τ=0.05 and τ=0.10 also yields positive $\Delta R_{OS}^{2}$ values and positive DM statistics, but these remain below conventional significance thresholds. For PLS, SHAP-PCA, and SHAP-PLS, the incremental gains are either small or unstable across thresholds. Taken together, the pairwise results indicate that the incremental contribution of forward-looking augmentation is selective rather than pervasive.

This pattern is consistent with the broader literature on aggregate equity premium prediction. A long line of work emphasizes that out-of-sample market return predictability is difficult to establish reliably, and that when predictability is present it is often weak and unstable in economic magnitude and statistical significance [1–3,20,55]. In this context, the limited pairwise DM significance in Table 6 should not be interpreted as evidence against the usefulness of the approach. Rather, it underscores the difficulty of extracting robust real-time signals from a single noisy aggregate return series. Our contribution is therefore not that forward-looking augmentation uniformly dominates past-only information, but that under disciplined admission and representation choices it can isolate weak but economically relevant predictive content, especially in specifications that also perform well in the downside-sensitive portfolio exercises. This interpretation is also in line with the view that poor out-of-sample *R*^2^ does not by itself rule out return predictability, and that even modest predictive improvements can matter economically [3,56].

#### 5.3.2 Sigma ablation study.

To make the role of the Stage 1 uncertainty channel explicit, Table 7 reports a direct sigma ablation for the combined feature representation at the representative interior admission threshold τ=0.10. The table compares each specification with and without the uncertainty proxy ${\hat{\sigma }}_{k,t+1|t}$, and reports the difference Δ in performance between the specification with ${\hat{\sigma }}_{k,t+1|t}$ and the corresponding specification without ${\hat{\sigma }}_{k,t+1|t}$. We focus on τ=0.10 in the main text to keep the ablation compact and directly interpretable, while the full τ-grid of the sigma ablation study is reported in S5 Appendix.

**Table 7:** Sigma ablation in Stage 2 for the combined features set at τ=0.10. Throughout the table, Δ denotes the difference in performance between the specification that includes the Stage 1 uncertainty proxy ${\hat{\sigma }}_{k,t+1|t}$ and the corresponding specification that excludes it. Panel A reports out-of-sample prediction metrics, and Panel B reports gross portfolio performance. Positive values of $\Delta R_{IS}^{2}$, $\Delta R_{OS}^{2}$, $\Delta R_{DOS}^{2}$, $\Delta R_{UOS}^{2}$, ΔSharpe, ΔSortino, and ΔCER indicate better performance with ${\hat{\sigma }}_{k,t+1|t}$, whereas negative values of ΔRRMSE and ΔMDD indicate improvement because forecast error and maximum drawdown are reduced.

*Panel A: Out-of-sample prediction metrics*					
Method	$\Delta R_{IS}^{2}$	$\Delta R_{OS}^{2}$	$\Delta R_{DOS}^{2}$	$\Delta R_{UOS}^{2}$	ΔRRMSE
–	−0.0003	0.0058	0.0197	−0.0065	−0.0029
PCA	0.0000	−0.0001	−0.0002	−0.0002	0.0000
PLS	0.0084	0.0136	0.0611	−0.0414	−0.0068
SHAP-PCA	0.0047	0.0103	0.0171	−0.0324	−0.0052
SHAP-PLS	0.0045	0.0143	0.0314	−0.0301	−0.0072
*Panel B: Portfolio performance*					
Method	ΔSharpe	ΔSortino	ΔCER	ΔMDD	ΔTurnover
–	0.0415	0.0614	0.0080	−0.0817	−0.0003
PCA	−0.0016	−0.0026	−0.0003	0.0000	−0.0007
PLS	0.1017	0.1566	0.0157	−0.0230	1.1540
SHAP-PCA	0.1546	0.2443	0.0302	−0.2130	0.3397
SHAP-PLS	0.1155	0.2044	0.0206	−0.1290	−0.0448

Panel A shows that the contribution of ${\hat{\sigma }}_{k,t+1|t}$ is not uniform across representations, but it is clearly not redundant. The cleanest comparison is the non-SHAP block, since it isolates the incremental role of the uncertainty proxy without additional screening effects. In the raw combined specification, adding sigma raises $R_{OS}^{2}$ and $R_{DOS}^{2}$, lowers $R_{UOS}^{2}$, and modestly improves RRMSE. Thus, even in the simplest non-SHAP specification, the uncertainty proxy improves forecasting performance primarily in adverse market states rather than uniformly across the test sample.

The PCA block, by contrast, is essentially unchanged. All prediction metrics remain close to zero, indicating that once the combined signal is compressed along variance-maximizing directions, the uncertainty channel adds little incremental information. This stands in clear contrast to the PLS block, which delivers the strongest forecasting gains without SHAP screening. There, sigma inclusion increases $R_{IS}^{2}$, $R_{OS}^{2}$, and $R_{DOS}^{2}$, but reduces $R_{UOS}^{2}$ while improving RRMSE. The asymmetry between $\Delta R_{DOS}^{2}$ and $\Delta R_{UOS}^{2}$ suggests that ${\hat{\sigma }}_{k,t+1|t}$ helps the second-stage learner identify states in which predictor-level forecasts are less precise and the mapping into the equity premium becomes more fragile or more nonlinear. In that sense, the uncertainty proxy functions as a state dependent reliability signal, which is precisely the role envisioned in Stage 1.

The SHAP-screened specifications reinforce this interpretation, but we treat them as complementary rather than primary evidence. Both SHAP-PCA and SHAP-PLS show positive changes in $R_{OS}^{2}$, and in both cases the improvement is again more pronounced on the downside than on the upside. These results are consistent with the non-SHAP evidence, but they should be interpreted with some caution because sigma inclusion may interact with screening and feature selection.

Panel B shows that the economic implications broadly mirror the statistical evidence. In the raw specification, sigma inclusion produces modest but consistently positive improvements in Sharpe, Sortino, and CER, while also reducing maximum drawdown. The PCA block again remains economically negligible. The strongest non-SHAP portfolio gains arise in the PLS block, where adding sigma improves the Sharpe ratio, the Sortino ratio, CER, and maximum drawdown, although turnover increases. Thus, in the representation where the gains are most visible, those gains also translate into a more attractive investment profile.

At the same time, the turnover results do not move in a single direction across methods. In particular, the PLS specification exhibits a higher turnover when sigma is included, whereas the raw and PCA specifications do not. This is useful for interpretation: The portfolio gains associated with ${\hat{\sigma }}_{k,t+1|t}$ are not simply the result of mechanically smoother portfolio weights or lower trading activity. Rather, they reflect an informational improvement in the signal entering the portfolio decision.

The SHAP-based portfolio results are again stronger in magnitude, with sizable gains in Sharpe, Sortino, CER, and drawdown control for both SHAP-PCA and SHAP-PLS. However, as in Panel A, we interpret these as robustness evidence rather than as the cleanest sigma comparison. The main takeaway from Table 7 is therefore not that sigma is universally helpful across all feature-processing choices. Instead, the evidence indicates that the Stage 1 uncertainty proxy has a distinct and economically meaningful role: its contribution is concentrated in downside-state forecasting, and it becomes most valuable when the combined signal is represented in a disciplined supervised low-dimensional form, especially PLS.

This interpretation is broadly in line with [41], who show that international economic policy uncertainty measures contain out-of-sample predictive information for U.S. excess returns, suggesting more generally that uncertainty related signals can be most useful when they are incorporated through a disciplined summary representation.

### 5.4 Interpretability: What drives predictability?

In this section, we study which economic channels are most closely associated with equity premium predictability over time by summarizing predictor importance at the group-level. Using the six predictor families defined in Section 4.1 (see Table 1 for the complete mapping), we compute mean absolute TreeSHAP attributions on the validation set and aggregate them within each family to obtain a time-varying measure of group-level importance. This aggregation helps mitigate multicollinearity induced attribution dilution among closely related predictors and yields a more stable, interpretable diagnostic of the channels emphasized by the forecasting model.

This group-level diagnostic also helps rationalize why SHAP-based screening improves out-of-sample performance. Because screening ranks predictors by their validation-based TreeSHAP contributions, it prioritizes variables that the fitted model relies on most when producing real-time forecasts, while de-emphasizing redundant signals within the same economic family. Consequently, the downstream forecasting stage is effectively guided toward the channels that carry predictive content at the forecast origin, which is consistent with the performance improvements documented in Tables 3–5.

Several broad patterns emerge. During the dot-com boom–bust (2000–2003), valuation signals are especially prominent, while issuance and financing variables are more visible than in later years, which is consistent with work linking valuation ratios and issuance waves to subsequent return reversals [57,58]. In the mid-2000s expansion, macroeconomic information gains relative importance, particularly around consumption–wealth conditions, while rates and credit variables remain an important backbone [18,59]. During the global financial crisis (2007–2009), the emphasis shifts more clearly toward rates/credit and market risk/comovement, in line with evidence on credit spreads, intermediary balance sheets, and tighter funding conditions [60,61]. Through much of the 2010s, market risk/comovement remains persistently important, and technical signals become more visible toward the late 2010s, which is consistent with the role of broad financial-cycle conditions and trend-sensitive information under prolonged monetary accommodation [21,22,62]. The 2020 pandemic shock again raises the relative importance of market risk/comovement together with rates/credit [63,64], while the 2022–2024 inflation-tightening period is characterized by renewed macroeconomic and rates/credit importance, with market risk/comovement remaining elevated rather than disappearing [65].

**Fig 1:**
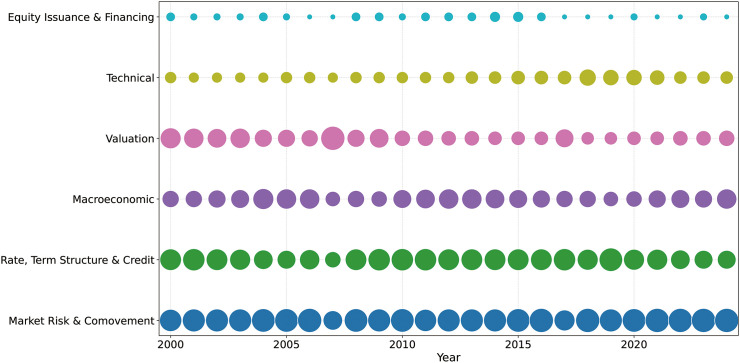
Time-varying group-level importance of equity premium predictor families. Each bubble corresponds to one of the six predictor families defined in Table 1 in a given calendar year. For each year, group-level importance is computed from mean absolute TreeSHAP values on the validation set and then aggregated within family. Bubble area reflects that family’s share of total annual group-level importance across the six families, so larger bubbles indicate that the fitted forecasting model relied more heavily on that family in that year.

This group-level view also helps make the role of SHAP-based screening easier to understand. The screening step is meant to keep the model focused on the predictors that are most informative at the forecast origin, while avoiding an unnecessarily wide set of nearby substitutes that often carry overlapping information. Read in that way, Fig 1 is not saying that one family causes the equity premium in a structural sense. Rather, it shows which kinds of signals the fitted forecasting system is leaning on most heavily in different periods. That is also why the family-level summary is useful: Even when the importance of individual variables moves around within a family, the broader economic channel can still remain visible in an interpretable way. Additionally, S6 Appendix examines, for a representative SHAP-PLS specification, how the composition of the annually selected SHAP-ranked predictor bundles evolves over time.

## 6 Conclusion

This paper studies real-time equity premium forecasting through a disciplined two-stage predict-the-predictors framework. Rather than mapping a high-dimensional and potentially unstable predictor panel directly into future excess returns, we first forecast each macro-financial predictor one step ahead and use its expected movement and forecast uncertainty to construct an augmented feature set. A predictor-level admission rule then determines whether these generated signals are sufficiently reliable to enter the second-stage forecasting model. Within this architecture, SHAP-based screening also serves as a useful layer of parsimony and interpretation, helping organize the augmented information in a more target-aligned way.

Empirically, the value of forward-looking augmentation is selective rather than uniform. Across representations and admission thresholds, reliability-screened forward-looking signals can improve benchmark relative forecasting performance and generate economically meaningful differences in portfolio outcomes, especially when predictive performance is examined through downside state diagnostics rather than average fit alone. The formal forecast comparison evidence is more supportive in benchmark relative comparisons than in stricter pairwise comparisons against matched past-only designs, which points to a nuanced but still meaningful contribution. The contribution of the paper is therefore not that combined inputs mechanically dominate past-only designs in every specification, but that forward-looking feature construction offers a disciplined way to extract incremental predictive and economic value from standard predictors when signal quality is screened, information is represented parsimoniously, and performance is evaluated in state-dependent as well as economic terms. The sigma ablation further suggests that the uncertainty channel is not redundant and can be especially informative in downside sensitive settings.

These findings also carry practical implications. For investors and asset allocators, they suggest that forward-looking transformations of standard predictors should be admitted selectively and judged not only by average forecast fit but also by their implications for downside protection, turnover, and implementable portfolio performance. For risk managers monitoring macro financial conditions, the framework offers a structured way to summarize which predictor families become more informative across regimes and when uncertainty related signals become more relevant. For policymakers and macro financial surveillance institutions, the framework may also serve as a descriptive tool for tracking when valuation, macroeconomic, credit, or market risk signals become more informative, which may be useful for regime monitoring and scenario-based risk assessment. In this sense, the paper contributes not only a forecasting design, but also a transparent bridge between real-time prediction, interpretable feature construction, and economic decision-making.

Several limitations point to natural next steps within the same empirical architecture. First, uncertainty is currently carried from Stage 1 to Stage 2 in a modular way. Bayesian or state space formulations could instead propagate predictive distributions more explicitly through the pipeline. Second, although we report net of transaction cost portfolio evidence, trading frictions are not yet embedded directly in model estimation or model selection. Future work could incorporate cost-aware validation rules or turnover sensitive objectives. Another extension is to examine alternative portfolio mappings, such as risk-parity allocation or utility specifications that place greater weight on downside outcomes, to assess whether the same forecasting signals remain valuable beyond the constrained mean–variance benchmark. Third, while our evaluation highlights downside state performance, the learning objective itself remains symmetric. Asymmetric, quantile-based, or downside-weighted losses could better align statistical learning with path-dependent investment goals. Finally, it would be valuable to examine the external validity of the framework beyond the present U.S. monthly setting, including emerging and frontier equity markets and, more ambitiously, higher-frequency settings such as mixed-frequency, intraday, or even tick-level environments in which information, frictions, and uncertainty evolve more rapidly. Relatedly, future work could extend to simple neural-network learners and richer meta-ensemble architectures, and could also allow dependence-aware first-stage models that exploit cross-predictor structure more explicitly.

## Supporting information

S1 Appendix Supplementary assessment of the stage 1 ARIMAX–GARCH specification. (PDF)

S2 Appendix Predictive robustness across alternative learners and equity universes. (PDF)

S3 Appendix Sensitivity of the tail-conditional *R*^2^ diagnostics to alternative quantile levels. (PDF)

S4 Appendix Robustness of portfolio performance to transaction costs and alternative volatility estimators. (PDF)

S5 Appendix Full sensitivity of the sigma ablation study. (PDF)

S6 Appendix Time variation in SHAP-ranked predictor bundles. (PDF)
