# Supplementary material for: Equity premium forecasting with reliability-screened forward-looking signals
Source: PLoS One. 2026 May 15;21(5):e0341578. doi: 10.1371/journal.pone.0341578 (PMC13178993; doi:10.1371/journal.pone.0341578)
Supplement: S1 Appendix — (PDF) [file pone.0341578.s001.pdf]

S1. Supplementary assessment of the stage 1 ARIMAX–GARCH specification

This appendix provides supplementary evidence on the Stage 1 ARIMAX–GARCH specification used to construct predictor-level forward-looking signals. Our objective is not to argue that a single first-stage model uniformly dominates every alternative for every predictor. Rather, because the predictor panel is economically heterogeneous and subject to real-time data constraints, we assess whether the chosen specification offers a reasonable balance of interpretability, cross-predictor comparability, and empirical stability in generating one-step-ahead conditional forecasts. The present section focuses on the robustness of the mean-forecast component of Stage 1. For compactness, the baseline ARIMAX results themselves are reported in Table 2 of the main text and are not repeated in Table S1, which reports only the alternative first-stage blocks used for comparison.

Table S1: **Mean-forecast comparison across alternative Stage 1 models.** This table reports predictor-level results for three alternative first-stage mean specifications evaluated under the same real-time data alignment and recursive one-step-ahead out-of-sample forecasting framework as the baseline ARIMAX procedure. The ARIMA block removes the exogenous component from the baseline specification, the ETS block reports results from a univariate additive Holt–Winters family selected by AICc, and the GPR block reports results from a supervised one-step-ahead Gaussian process model based on low-order autoregressive lags and, when included, a single publication-lag-adjusted exogenous predictor. Within each model block, predictors are sorted in descending order of out-of-sample R^2_{OS}.

ARIMA				ETS				GPR			
Variable	Order	R^2_{IS}	R^2_{OS}	Variable	Model	R^2_{IS}	R^2_{OS}	Variable	Exo. vars	R^2_{IS}	R^2_{OS}
ik	(2,0,0)	0.2363	0.2776	infl	ANA	0.4193	0.2058	ik	—	0.4580	0.1803
de	(2,0,0)	0.4123	0.2470	de	AAdN	0.3980	0.1776	infl	bm	0.4935	0.1294
svar	(1,0,0)	0.2985	0.2134	svar	ANN	0.2472	0.1489	tbl	dy	0.3433	0.1110
avgcor	(1,0,2)	0.2368	0.1854	tail	ANN	0.0910	0.0868	avgcor	tbl	0.3535	0.1110
dfy	(1,0,1)	0.1347	0.1102	cay	ANN	0.1233	0.0299	tail	eqis	0.4595	0.0826
tchi	(1,0,1)	0.1267	0.0890	bm	ANA	0.0776	0.0202	de	tchi	0.7935	0.0771
tail	(1,0,1)	0.1006	0.0861	gpce	ANN	-0.0154	0.0049	tchi	dy	0.3350	0.0729
infl	(2,0,2)	0.3155	0.0706	avgcor	ANN	-0.0000	-0.0000	ogap	tchi	0.4351	0.0643
tbl	(2,0,0)	0.1262	0.0619	tchi	ANN	-0.0000	-0.0000	dfy	dp	0.5075	0.0581
ogap	(2,0,0)	0.1086	0.0354	dp	ANN	-0.0004	-0.0004	tms	ltr	0.2123	0.0433
tms	(2,0,0)	0.0367	0.0045	dy	ANN	-0.0004	-0.0004	gpce	avgcor	0.6512	0.0389
lty	(2,0,1)	0.0071	0.0040	tms	ANN	-0.0000	-0.0005	ep	tms	0.3351	0.0328
dy	(0,1,0)	0.0000	0.0000	ep	ANN	-0.0000	-0.0009	ntis	de	0.3169	0.0087
ep	(0,1,0)	0.0000	0.0000	ltr	ANN	-0.0003	-0.0009	cay	dfr	0.7351	0.0028
dp	(0,1,0)	0.0000	-0.0000	ntis	ANN	-0.0000	-0.0012	dy	ltr	0.4694	-0.0038
ntis	(1,0,0)	0.0094	-0.0071	lty	ANN	-0.0000	-0.0019	eqis	dfy	0.6776	-0.0049
bm	(1,0,0)	0.0022	-0.0156	dfy	ANN	-0.0030	-0.0031	svar	ltr	0.4616	-0.0092
ltr	(2,0,2)	0.0078	-0.0217	ik	ANN	-0.0045	-0.0053	dp	ogap	0.3852	-0.0209
dfr	(1,0,1)	0.0131	-0.0247	ogap	AAdN	0.0654	-0.0083	bm	ogap	0.2127	-0.0263
gpce	(2,0,0)	0.0366	-0.0784	dfr	ANA	0.0250	-0.0143	dfr	svar	0.3008	-0.0422
cay	(1,0,1)	0.1488	-0.0821	tbl	AAdN	0.0003	-0.0328	ltr	tms	0.3614	-0.0453
eqis	(0,0,0)	-0.7680	-1.4231	eqis	ANN	0.0725	-0.1900	lty	svar	0.1941	-0.0585

Mean-Forecast Robustness of the Stage 1 ARIMAX Specification. Table S1 compares the baseline Stage 1 ARIMAX mean specification with three alternative first-stage forecasting models: a univariate ARIMA benchmark, an exponential-smoothing (ETS) benchmark, and a Gaussian process regression (GPR) benchmark. For comparability, all alternatives are evaluated under the same real-time data alignment and recursive one-step-ahead out-of-sample forecasting framework used in the baseline procedure. The ARIMA benchmark is obtained by removing the exogenous block from the baseline specification while preserving the same forecasting design. The ETS benchmark is implemented as a deliberately parsimonious uni-

variate alternative: for each predictor, we estimate a restricted family of additive Holt–Winters specifications—ANN, AAN, and damped AAdN, together with additive seasonal variants when the data frequency permits seasonality—and select the preferred specification using the Akaike information criterion with finite-sample correction (AICc). The GPR benchmark is implemented as a supervised one-step-ahead forecasting model based on low-order autoregressive lags and, when included, a single publication-lag-adjusted exogenous predictor. In its baseline implementation, the GPR model uses two autoregressive lags, standardized inputs, a Matern(5/2) kernel with a white-noise component, an automatic choice between level and difference forecasting depending on the target variable, and a capped training window for computational tractability. Whenever exogenous information is used, it is aligned using the same real-time publication-lag rules and subject to the same exclusion restrictions as in the baseline Stage 1 procedure. These alternatives are therefore intended as disciplined and interpretable benchmarks rather than aggressively tuned black-box competitors.

The results in Table S1 show that simple autoregressive dynamics remain competitive for several of the more persistent predictors, indicating that an important share of predictive content is contained in own-series behavior. This pattern is consistent with the broader return-predictability literature, where commonly used financial state variables—such as dividend yield, book-to-market, and earnings-price ratios—are routinely treated as highly persistent autoregressive processes (Stambaugh, 1999; Lewellen, 2004; Campbell and Yogo, 2006). At the same time, the ARIMAX specification improves upon the univariate benchmark for a nontrivial subset of predictors, especially among selected macroeconomic and rate/spread-related variables, suggesting that cross-predictor information can add value when incorporated in a disciplined and comparable manner under real-time alignment and exclusion restrictions. By contrast, ETS and GPR deliver gains only for selected series and do not uniformly dominate the ARIMAX benchmark across the full predictor panel. This broader pattern is also in line with large-scale forecasting evidence showing that parsimonious linear autoregressive models remain strong benchmarks across many macroeconomic time series (Marcellino et al., 2006), as well as with inflation-forecasting evidence that simple univariate time-series structures continue to provide informative baselines (Stock and Watson, 2007). Taken together, these findings support the Stage 1 ARIMAX specification as a balanced first-stage device: it is parsimonious, interpretable, and broadly competitive, while still allowing targeted gains from carefully selected exogenous information.

Calibration of the Stage 1 GARCH-Based Volatility Proxy. Table S2 shifts the focus from the mean side of Stage 1 to the calibration of the uncertainty signal, while holding the Stage 1 ARIMAX conditional mean fixed throughout. Specifically, we compare the baseline GARCH-based conditional volatility with a simpler constant-volatility benchmark constructed from the same ARIMAX mean specification. For the latter, at each recursive forecast origin we estimate the residual variance from the corresponding expanding-window ARIMAX mean regression and use its square root as a homoskedastic next-period volatility proxy. This design provides a clean test of whether the GARCH layer adds value beyond a residual-based constant-volatility benchmark, because the underlying one-step-ahead mean forecast is identical across the two blocks and only the volatility proxy is varied.

For each predictor, Vol. reports the square root of the average out-of-sample predicted variance, i.e., the forecasted volatility scale implied by the corresponding proxy. RMSE reports the root mean squared out-of-sample mean-forecast error and therefore provides the realized forecast-error scale; by construction, it is the same across the two blocks because the mean forecast is held fixed. Ratio is defined as the ratio of Vol. to RMSE, so values closer to one indicate that the forecasted volatility scale is more closely aligned with the realized forecast-error scale. Cal. Error is the absolute deviation of this ratio from one and therefore summarizes the extent of miscalibration in scale terms. We also report the QLIKE loss, computed from the realized squared forecast error and the predicted conditional variance following Patton (2011), which is standard in volatility-forecast evaluation; lower values indicate a more informative and better calibrated volatility proxy.

The evidence in Table S2 favors the GARCH-based specification. Across the 22 predictors, the GARCH proxy delivers a lower QLIKE value for 19 variables and a smaller calibration error for 19 variables. The cross-predictor average Ratio is 1.006 under GARCH, compared with 1.178 under the constant-volatility

Table S2: **Out-of-sample calibration results for two alternative Stage 1 volatility proxies constructed from the same ARIMAX conditional mean forecasts.** The left block reports a constant-volatility benchmark, defined as the square root of the residual variance from the corresponding expanding-window ARIMAX mean regression. The right block reports the baseline GARCH-based conditional volatility. For each predictor, Vol. is defined as the square root of the mean out-of-sample predicted conditional variance across forecast origins. RMSE is defined as the root mean squared out-of-sample one-step-ahead mean-forecast error. Ratio is defined as Vol./RMSE. Cal. Error is defined as the absolute deviation of Ratio from one. QLIKE is computed from the realized squared out-of-sample forecast error and the predicted conditional variance following Patton (2011). Lower values of Cal. Error and QLIKE indicate better calibration.

	Constant volatility					GARCH				
Variable	Vol.	RMSE	Ratio	Cal. Error	QLIKE	Vol.	RMSE	Ratio	Cal. Error	QLIKE
`eqis`	0.0606	0.0590	1.0257	0.0257	-4.6435	0.0585	0.0590	0.9911	0.0089	-4.5962
`gpce`	0.0053	0.0044	1.1938	0.1938	-9.7812	0.0051	0.0044	1.1587	0.1587	-9.7986
`ik`	0.0008	0.0006	1.2902	0.2902	-13.6146	0.0008	0.0006	1.1855	0.1855	-13.7162
`cay`	0.0077	0.0214	0.3606	0.6394	-2.8526	0.0150	0.0214	0.7024	0.2976	-6.3490
`ep`	0.0057	0.0035	1.6633	0.6633	-10.0236	0.0034	0.0035	0.9931	0.0069	-10.5367
`dp`	0.0031	0.0014	2.2205	1.2205	-11.3949	0.0017	0.0014	1.1878	0.1878	-12.1651
`dy`	0.0030	0.0013	2.3318	1.3318	-11.5117	0.0015	0.0013	1.1691	0.1691	-12.3077
`ntis`	0.0035	0.0039	0.9123	0.0877	-10.0427	0.0037	0.0039	0.9422	0.0578	-10.0379
`svar`	0.0030	0.0051	0.5852	0.4148	-3.5885	0.0036	0.0051	0.6955	0.3045	-3.2790
`dfr`	0.0123	0.0151	0.8110	0.1890	-7.3257	0.0151	0.0151	0.9944	0.0056	-7.7253
`bm`	0.0419	0.0258	1.6254	0.6254	-6.0638	0.0312	0.0258	1.2104	0.2104	-6.3859
`tms`	0.0041	0.0042	0.9866	0.0134	-9.7865	0.0044	0.0042	1.0492	0.0492	-10.2935
`lty`	0.0078	0.0031	2.4979	1.4979	-10.5312	0.0037	0.0031	1.1978	0.1978	-10.6954
`dfy`	0.0010	0.0011	0.8581	0.1419	-12.3017	0.0011	0.0011	0.9717	0.0283	-13.0540
`tchi`	0.5775	0.5671	1.0184	0.0184	-0.1241	0.6262	0.5671	1.1041	0.1041	-0.2559
`tbl`	0.0044	0.0041	1.0658	0.0658	-9.8498	0.0042	0.0041	1.0032	0.0032	-11.1389
`ogap`	0.0098	0.0094	1.0333	0.0333	-8.2964	0.0095	0.0094	1.0047	0.0047	-8.6784
`infl`	0.0027	0.0031	0.8894	0.1106	-10.5337	0.0029	0.0031	0.9404	0.0596	-10.6657
`de`	0.0679	0.0726	0.9351	0.0649	-1.7951	0.0559	0.0726	0.7696	0.2304	-7.6514
`ltr`	0.0252	0.0307	0.8204	0.1796	-5.8041	0.0295	0.0307	0.9605	0.0395	-6.0096
`tail`	0.0157	0.0176	0.8935	0.1065	-7.0732	0.0162	0.0176	0.9224	0.0776	-7.0901
`avgcor`	0.0447	0.0495	0.9035	0.0965	-4.9935	0.0489	0.0495	0.9881	0.0119	-5.0620

benchmark, indicating that the GARCH-implied volatility scale is, on average, much closer to the realized out-of-sample error scale. Likewise, the average Cal. Error declines from 0.364 under the constant-volatility benchmark to 0.109 under GARCH, and the average QLIKE decreases from -7.815 to -8.522. Although a small minority of predictors still favor the constant-volatility benchmark under one metric or the other, the overall pattern is clear: Allowing the volatility proxy to vary conditionally through GARCH yields more tightly calibrated predictor-level uncertainty measures than the constant-volatility benchmark derived from the same ARIMAX mean specification.

Taken together, these findings suggest that the GARCH component in Stage 1 is not merely a conventional add-on to the ARIMAX mean equation. Rather, it provides a practically useful uncertainty signal that is more tightly aligned with realized forecast uncertainty than a constant-volatility benchmark derived from the same mean specification. This strengthens the interpretation of $\hat{\sigma}_{k,t+1|t}$ in Stage 2: it is not simply an auxiliary feature, but a forward-looking measure of local forecast uncertainty whose empirical calibration is supported by the data. Combined with the mean-forecast robustness results reported above, the evidence in this appendix supports the Stage 1 ARIMAX-GARCH specification as a parsimonious, interpretable, and empirically well-calibrated first-stage device for generating comparable predictor-level signals under real-time constraints.

References

Campbell, J. Y. and Yogo, M. (2006). Efficient tests of stock return predictability. *Journal of financial economics*, 81(1):27–60.

Lewellen, J. (2004). Predicting returns with financial ratios. *Journal of financial economics*, 74(2):209–235.

Marcellino, M., Stock, J. H., and Watson, M. W. (2006). A comparison of direct and iterated multistep ar methods for forecasting macroeconomic time series. *Journal of econometrics*, 135(1-2):499–526.

Patton, A. J. (2011). Volatility forecast comparison using imperfect volatility proxies. *Journal of econometrics*, 160(1):246–256.

Stambaugh, R. F. (1999). Predictive regressions. *Journal of financial economics*, 54(3):375–421.

Stock, J. H. and Watson, M. W. (2007). Why has us inflation become harder to forecast? *Journal of Money, Credit and banking*, 39:3–33.
