# Supplementary material for: Equity premium forecasting with reliability-screened forward-looking signals
Source: PLoS One. 2026 May 15;21(5):e0341578. doi: 10.1371/journal.pone.0341578 (PMC13178993; doi:10.1371/journal.pone.0341578)
Supplement: S2 Appendix — (PDF) [file pone.0341578.s002.pdf]

S2. Predictive robustness across alternative learners and equity universes

This appendix reports two complementary robustness exercises. First, Tables S3–S6 replace the baseline Random Forest learner in Stage 2 with LightGBM and XGBoost while preserving the same feature definitions, admission-threshold grid, expanding-window estimation protocol, and evaluation metrics used in the main text. Second, Tables S7–S8 repeat the Stage 2 exercise for the CRSP value-weighted index under the same training, validation, and test windows as in the baseline S&P 500 analysis.

Table S3: **Portfolio performance using LightGBM, by feature family and estimation method.** The table reports Sharpe ratio, Sortino ratio, certainty equivalent return (CER) with risk aversion $\gamma = 3$, maximum drawdown (MDD), and turnover for various forecasting strategies. The reliability threshold τ refers to the minimum individual predictor out-of-sample R^2 from Stage 1 required for its forward-looking forecast to be included in the combined feature set.

Method	Feature	τ	Sharpe	Sortino	CER	MDD	Turnover
Buy & Hold			0.4560	0.6626	0.0347	0.5022	–
CAPM	Past	–	0.2921	0.3949	0.0109	0.5127	2.7710
FF3			0.2600	0.3606	0.0025	0.5936	5.3486
–	Past	–	0.2933	0.3897	0.0083	0.6079	1.0703
	Comb.	0.00	0.3039	0.4090	0.0095	0.5545	1.1663
		0.05	0.3460	0.4671	0.0157	0.6205	1.3384
		0.10	0.3914	0.5416	0.0231	0.5939	1.3374
		0.15	0.3280	0.4423	0.0125	0.5545	1.4649
		0.20	0.2658	0.3538	0.0033	0.5552	1.0071
PCA	Past	–	0.3160	0.4399	0.0107	0.6645	0.8002
	Comb.	0.00	0.3302	0.4627	0.0125	0.6500	0.7406
		0.05	0.3360	0.4731	0.0135	0.6592	0.7237
		0.10	0.3270	0.4570	0.0121	0.6611	0.7706
		0.15	0.2710	0.3652	0.0039	0.6630	0.7996
		0.20	0.2936	0.3976	0.0083	0.6636	0.6449
PLS	Past	–	0.5195	0.7507	0.0449	0.6317	1.7680
	Comb.	0.00	0.2520	0.3373	0.0016	0.5513	2.0089
		0.05	0.4465	0.6272	0.0332	0.4420	2.5785
		0.10	0.5356	0.7650	0.0462	0.5127	2.5432
		0.15	0.5543	0.8336	0.0462	0.3952	2.7784
		0.20	0.6198	0.9793	0.0554	0.3403	3.1125
SHAP-PCA	Past	–	0.5339	0.7979	0.0471	0.3016	2.6894
	Comb.	0.00	0.4069	0.5629	0.0261	0.6086	0.9833
		0.05	0.4614	0.6516	0.0355	0.5023	1.9431
		0.10	0.4498	0.6266	0.0331	0.6241	1.8641
		0.15	0.4984	0.7186	0.0414	0.4992	1.7321
		0.20	0.4823	0.7028	0.0387	0.6127	1.5383
SHAP-PLS	Past	–	0.5459	0.8203	0.0493	0.3477	3.2332
	Comb.	0.00	0.5380	0.8220	0.0468	0.2847	2.3392
		0.05	0.5823	0.8779	0.0559	0.5064	1.8106
		0.10	0.6714	1.0481	0.0668	0.2844	2.2707
		0.15	0.7945	1.3412	0.0817	0.2736	3.4260
		0.20	0.4704	0.6864	0.0369	0.5517	1.9783

Robustness across alternative learners. Tables S3–S6 broadly reinforce the main-text message that forward-looking augmentation is representation-dependent rather than mechanically beneficial. Under both Light-

Table S4: **Out-of-sample R^2 for the equity risk premium forecasts using LightGBM.** The table reports the in-sample R^2 and three out-of-sample R^2 measures relative to the historical mean benchmark, by feature family, estimation method, and predictor-level threshold τ. The threshold τ refers to the minimum individual predictor out-of-sample R^2 from Stage 1 required for its forward-looking forecast to be included in the combined feature set. R^2_{DOS} and R^2_{UOS} denote out-of-sample R^2 in downside (left-tail) and upside (right-tail) months, as defined in Section 3.3.

Method	Feature	τ	R^2_{IS}	R^2_{OS}	R^2_{DOS}	R^2_{UOS}	RRMSE
–	Past	–	0.0337	−0.0056	−0.0223	0.0052	1.0028
	Comb.	0.00	0.0437	−0.0071	−0.0210	0.0064	1.0035
		0.05	0.0421	−0.0018	−0.0324	0.0219	1.0009
		0.10	0.0419	0.0009	−0.0307	0.0311	0.9995
		0.15	0.0429	−0.0091	−0.0353	0.0125	1.0045
		0.20	0.0321	−0.0092	−0.0217	0.0027	1.0046
PCA	Past	–	0.0256	−0.0026	−0.0191	0.0227	1.0013
	Comb.	0.00	0.0258	−0.0016	−0.0195	0.0283	1.0008
		0.05	0.0253	0.0025	−0.0272	0.0402	0.9987
		0.10	0.0240	0.0023	−0.0218	0.0306	0.9989
		0.15	0.0247	−0.0019	−0.0147	0.0137	1.0009
		0.20	0.0235	0.0007	−0.0020	0.0112	0.9996
PLS	Past	–	0.0560	0.0012	−0.0071	−0.0020	0.9994
	Comb.	0.00	0.0409	−0.0094	−0.0022	−0.0007	1.0047
		0.05	0.0697	−0.0010	0.0535	−0.0749	1.0005
		0.10	0.0692	0.0042	0.0585	−0.0711	0.9979
		0.15	0.0682	−0.0058	0.0947	−0.1268	1.0029
		0.20	0.0657	−0.0074	0.0944	−0.1235	1.0037
SHAP-PCA	Past	–	0.0318	0.0135	0.0188	0.0405	0.9932
	Comb.	0.00	0.0244	0.0044	−0.0247	0.0128	0.9978
		0.05	0.0378	0.0113	0.0037	0.0165	0.9944
		0.10	0.0331	0.0117	−0.0250	0.0342	0.9941
		0.15	0.0342	0.0159	0.0143	0.0253	0.9920
		0.20	0.0286	0.0125	−0.0096	0.0385	0.9937
SHAP-PLS	Past	–	0.0491	0.0137	−0.0061	0.0388	0.9931
	Comb.	0.00	0.0430	0.0043	0.0475	−0.0161	0.9978
		0.05	0.0426	0.0217	−0.0156	0.0597	0.9891
		0.10	0.0639	0.0158	0.0433	−0.0232	0.9921
		0.15	0.0590	0.0193	0.0833	−0.0706	0.9903
		0.20	0.0346	0.0049	0.0368	−0.0178	0.9975

GBM and XGBoost, the unreduced Combined block remains weak in aggregate out-of-sample fit, and the unsupervised PCA block delivers only modest gains at selected thresholds. By contrast, stronger results arise when the admitted forward-looking signals are paired with outcome-aligned representations, especially PLS-based and SHAP-screened specifications. For LightGBM, SHAP-PLS Combined reaches $R^2_{OS} = 0.0217$ at $\tau = 0.05$ and a Sharpe ratio of 0.7945 at $\tau = 0.15$. For XGBoost, SHAP-PLS Combined attains $R^2_{OS} = 0.0192$ at $\tau = 0.10$ and a Sharpe ratio of 0.6546 at $\tau = 0.15$.

These robustness checks also preserve the same threshold and tail-state interpretation emphasized in Section 5.2. Tightening τ should not be interpreted as a monotone tuning rule: Under both boosting learners, performance typically improves only at selected interior thresholds and can weaken again under tighter admission. Likewise, the tail-conditional diagnostics continue to show a meaningful representation contrast. PCA-type specifications remain relatively more aligned with upside accuracy, whereas PLS-type

Combined specifications more often concentrate gains in downside states. Taken together, Tables S3–S6 suggest that the same substantive conclusion continues to hold: forward-looking augmentation can add value, but mainly when the generated signals are admitted selectively and passed through representations that preserve return-relevant directions.

Table S5: **Portfolio performance using XGBoost, by feature family and estimation method.** The table reports Sharpe ratio, Sortino ratio, certainty equivalent return (CER) with risk aversion $\gamma = 3$, maximum drawdown (MDD), and turnover for various forecasting strategies. The reliability threshold τ refers to the minimum individual predictor out-of-sample R^2 from Stage 1 required for its forward-looking forecast to be included in the combined feature set.

Method	Feature	τ	Sharpe	Sortino	CER	MDD	Turnover
Buy & Hold			0.4560	0.6626	0.0347	0.5022	–
CAPM	Past	–	0.2921	0.3949	0.0109	0.5127	2.7710
FF3			0.2600	0.3606	0.0025	0.5936	5.3486
–	Past	–	0.2822	0.3757	0.0067	0.5893	0.7002
	Comb.	0.00	0.2918	0.3885	0.0087	0.5769	0.6453
		0.05	0.3271	0.4435	0.0135	0.5705	0.6385
		0.10	0.3126	0.4220	0.0109	0.5973	0.6339
		0.15	0.2982	0.3984	0.0086	0.6216	0.6852
		0.20	0.2800	0.3731	0.0063	0.5536	0.7440
PCA	Past	–	0.2942	0.4032	0.0072	0.6645	0.6108
	Comb.	0.00	0.2944	0.4023	0.0069	0.6647	0.5777
		0.05	0.3210	0.4427	0.0115	0.6643	0.6181
		0.10	0.3105	0.4285	0.0095	0.6645	0.5885
		0.15	0.2871	0.3899	0.0067	0.6645	0.5423
		0.20	0.2872	0.3880	0.0074	0.6457	0.5151
PLS	Past	–	0.4693	0.6606	0.0366	0.6518	1.6096
	Comb.	0.00	0.2684	0.3574	0.0058	0.5374	1.6146
		0.05	0.4045	0.5589	0.0271	0.4961	2.3503
		0.10	0.4807	0.6691	0.0383	0.5463	2.1555
		0.15	0.4610	0.6602	0.0349	0.5045	2.4939
		0.20	0.5645	0.8642	0.0486	0.3486	2.3080
SHAP-PCA	Past	–	0.4212	0.5912	0.0290	0.6336	1.9800
	Comb.	0.00	0.4664	0.6796	0.0363	0.4892	1.5483
		0.05	0.3830	0.5342	0.0218	0.6359	1.2316
		0.10	0.4285	0.6049	0.0291	0.6645	0.7045
		0.15	0.3615	0.4940	0.0182	0.6645	0.7923
		0.20	0.3879	0.5399	0.0225	0.6550	0.5936
SHAP-PLS	Past	–	0.4399	0.6230	0.0317	0.6615	1.9173
	Comb.	0.00	0.4325	0.6488	0.0311	0.5103	1.8147
		0.05	0.5400	0.7987	0.0477	0.4547	1.7595
		0.10	0.5774	0.8531	0.0541	0.4189	2.1109
		0.15	0.6546	1.0024	0.0648	0.5207	2.0291
		0.20	0.4449	0.6235	0.0329	0.5625	1.8759

Robustness across equity universes. Tables S7–S8 show that the main qualitative patterns also carry over to the CRSP value-weighted index, although the magnitudes are somewhat more muted than in the baseline S&P 500 results. The unreduced Combined block remains generally weak, and the benefits of augmentation again depend on how the feature pool is represented before entering the learner. Among the statistical

Table S6: **Out-of-sample R^2 for the equity risk premium forecasts using XGBoost.** The table reports the in-sample R^2 and three out-of-sample R^2 measures relative to the historical mean benchmark, by feature family, estimation method, and predictor-level threshold τ. The threshold τ refers to the minimum individual predictor out-of-sample R^2 from Stage 1 required for its forward-looking forecast to be included in the combined feature set. R^2_{DOS} and R^2_{UOS} denote out-of-sample R^2 in downside (left-tail) and upside (right-tail) months, as defined in Section 3.3.

Method	Feature	τ	R^2_{IS}	R^2_{OS}	R^2_{DOS}	R^2_{UOS}	RRMSE
–	Past	–	0.0209	−0.0054	−0.0178	0.0049	1.0027
	Comb.	0.00	0.0215	−0.0040	−0.0112	0.0033	1.0020
		0.05	0.0216	−0.0021	−0.0157	0.0126	1.0010
		0.10	0.0221	−0.0030	−0.0200	0.0153	1.0015
		0.15	0.0222	−0.0035	−0.0216	0.0158	1.0018
		0.20	0.0205	−0.0058	−0.0145	0.0039	1.0029
PCA	Past	–	0.0176	−0.0034	−0.0163	0.0179	1.0017
	Comb.	0.00	0.0175	−0.0022	−0.0167	0.0200	1.0011
		0.05	0.0175	0.0012	−0.0183	0.0238	0.9994
		0.10	0.0164	0.0015	−0.0152	0.0222	0.9992
		0.15	0.0179	−0.0000	−0.0120	0.0163	1.0000
		0.20	0.0170	−0.0007	−0.0040	0.0072	1.0003
PLS	Past	–	0.0437	0.0034	−0.0076	0.0021	0.9983
	Comb.	0.00	0.0300	−0.0065	0.0032	−0.0082	1.0033
		0.05	0.0528	0.0056	0.0315	−0.0410	0.9972
		0.10	0.0524	0.0050	0.0348	−0.0485	0.9975
		0.15	0.0519	0.0048	0.0604	−0.0683	0.9976
		0.20	0.0484	0.0029	0.0729	−0.0772	0.9985
SHAP-PCA	Past	–	0.0226	0.0082	0.0048	0.0174	0.9959
	Comb.	0.00	0.0218	0.0094	0.0014	0.0239	0.9953
		0.05	0.0178	0.0050	−0.0190	0.0306	0.9975
		0.10	0.0200	0.0086	−0.0303	0.0435	0.9957
		0.15	0.0236	0.0032	−0.0309	0.0302	0.9984
		0.20	0.0188	0.0049	−0.0222	0.0213	0.9975
SHAP-PLS	Past	–	0.0264	0.0125	0.0032	0.0266	0.9937
	Comb.	0.00	0.0358	0.0156	0.0486	−0.0076	0.9922
		0.05	0.0313	0.0124	0.0241	−0.0052	0.9938
		0.10	0.0429	0.0192	0.0111	0.0025	0.9904
		0.15	0.0398	0.0139	0.0169	−0.0174	0.9930
		0.20	0.0379	0.0046	0.0164	−0.0328	0.9977

results, SHAP-based specifications remain the most consistently positive. SHAP-PCA Combined reaches $R^2_{OS} = 0.0158$ at $\tau = 0.15$, while SHAP-PLS Past already attains $R^2_{OS} = 0.0230$ and the Combined variant remains competitive at $\tau = 0.05$ (0.0236). PLS-based Combined designs can also improve selected configurations, but the threshold effect is again specification-dependent rather than monotone.

The portfolio results point in the same direction. No single Combined specification uniformly dominates its Past counterpart, yet selected configurations continue to yield economically meaningful improvements. For example, SHAP-PLS Combined reaches a Sharpe ratio of 0.5830 at $\tau = 0.05$, exceeding the SHAP-PLS Past benchmark (0.4977), while SHAP-PCA Combined reaches 0.4768 at $\tau = 0.20$ relative to 0.4452 for SHAP-PCA Past. The conditional diagnostics remain informative here as well: PCA-type specifications tend to preserve relatively stronger upside alignment, whereas PLS-type Combined specifications more often improve downside fit. Overall, Tables S7–S8 support a robustness interpretation rather than a

Table S7: **Portfolio performance based on the CRSP value-weighted index by feature family and estimation method.** The table reports Sharpe ratio, Sortino ratio, certainty equivalent return (CER) with risk aversion $\gamma = 3$, maximum drawdown (MDD), and turnover for various forecasting strategies. The reliability threshold τ refers to the minimum individual predictor out-of-sample R^2 from Stage 1 required for its forward-looking forecast to be included in the combined feature set.

Method	Feature	τ	Sharpe	Sortino	CER	MDD	Turnover
Buy & Hold			0.4142	0.5910	0.0280	0.5148	–
CAPM	Past	–	0.1877	0.2414	−0.0052	0.5648	4.4715
FF3			0.2875	0.3796	0.0078	0.5792	5.6588
–	Past	–	0.1965	0.2505	−0.0067	0.6077	0.8899
	Comb.	0.00	0.1641	0.2063	−0.0099	0.5862	0.7052
		0.05	0.1799	0.2282	−0.0095	0.5862	0.8376
		0.10	0.2792	0.3693	0.0050	0.6117	0.7068
		0.15	0.2375	0.3077	−0.0009	0.6006	0.9628
		0.20	0.1912	0.2433	−0.0068	0.5783	0.9830
PCA	Past	–	0.2671	0.3670	0.0040	0.6749	0.6153
	Comb.	0.00	0.3124	0.4176	0.0115	0.5579	0.9566
		0.05	0.2865	0.3931	0.0057	0.6744	0.8112
		0.10	0.3344	0.4479	0.0146	0.5365	0.8598
		0.15	0.2147	0.2807	−0.0052	0.6764	0.7328
		0.20	0.2393	0.3154	0.0002	0.6682	0.7344
PLS	Past	–	0.5159	0.7646	0.0443	0.6789	2.3086
	Comb.	0.00	0.5223	0.7737	0.0425	0.4979	1.8343
		0.05	0.4713	0.6665	0.0368	0.5014	2.2383
		0.10	0.4337	0.6442	0.0313	0.4303	2.6642
		0.15	0.4174	0.5901	0.0290	0.5798	2.1476
		0.20	0.4670	0.7031	0.0354	0.5053	2.9084
SHAP-PCA	Past	–	0.4452	0.6310	0.0328	0.5105	1.4996
	Comb.	0.00	0.3694	0.5211	0.0169	0.5983	1.5340
		0.05	0.3905	0.5412	0.0234	0.5589	2.2023
		0.10	0.3611	0.4936	0.0183	0.6672	1.4047
		0.15	0.4158	0.5722	0.0286	0.4781	2.2905
		0.20	0.4768	0.7256	0.0376	0.4642	2.1195
SHAP-PLS	Past	–	0.4977	0.7427	0.0412	0.4267	1.2967
	Comb.	0.00	0.3875	0.5303	0.0249	0.5132	1.8308
		0.05	0.5830	0.8789	0.0543	0.3393	1.6661
		0.10	0.4725	0.6661	0.0372	0.4621	1.7761
		0.15	0.3671	0.5121	0.0216	0.3191	1.3430
		0.20	0.5260	0.8011	0.0435	0.3546	2.9926

claim of universal dominance. In a different broad market equity universe, the gains from forward-looking augmentation remain selective, representation-dependent, and sensitive to the predictor-level admission rule.

Table S8: **Out-of-sample R^2 for CRSP value-weighted index excess return forecasts by feature family, estimation method, and predictor-level threshold τ.** The table reports the in-sample R^2 and three out-of-sample R^2 measures relative to historical expanding mean benchmark. The threshold τ refers to the minimum individual predictor out-of-sample R^2 from Stage 1 required for its forward-looking forecast to be included in the combined feature set. R^2_{DOS} and R^2_{UOS} denote out-of-sample R^2 in downside (left-tail) and upside (right-tail) months, as defined in Section 3.3.

Method	Feature	τ	R^2_{IS}	R^2_{OS}	R^2_{DOS}	R^2_{UOS}	RRMSE
–	Past	–	0.0241	−0.0077	−0.0190	−0.0018	1.0038
	Comb.	0.00	0.0229	−0.0091	−0.0104	−0.0134	1.0045
		0.05	0.0259	−0.0112	−0.0252	−0.0019	1.0056
		0.10	0.0207	−0.0001	−0.0208	0.0251	1.0001
		0.15	0.0232	−0.0024	−0.0207	0.0190	1.0012
		0.20	0.0226	−0.0067	−0.0127	−0.0022	1.0034
PCA	Past	–	0.0206	0.0014	−0.0059	0.0144	0.9993
	Comb.	0.00	0.0180	0.0015	−0.0131	0.0189	0.9993
		0.05	0.0190	0.0033	−0.0163	0.0306	0.9983
		0.10	0.0223	0.0018	−0.0137	0.0212	0.9991
		0.15	0.0200	−0.0008	−0.0153	0.0149	1.0004
		0.20	0.0190	0.0012	−0.0006	0.0060	0.9994
PLS	Past	–	0.0455	0.0054	−0.0044	0.0295	0.9973
	Comb.	0.00	0.0510	0.0079	0.0810	−0.0779	0.9961
		0.05	0.0567	0.0023	0.0482	−0.0490	0.9988
		0.10	0.0566	0.0061	0.0350	−0.0209	0.9969
		0.15	0.0563	0.0011	0.0724	−0.0831	0.9995
		0.20	0.0587	0.0001	0.0658	−0.0869	0.9999
SHAP-PCA	Past	–	0.0309	0.0104	0.0044	0.0138	0.9948
	Comb.	0.00	0.0140	0.0048	−0.0507	0.0766	0.9976
		0.05	0.0229	0.0128	−0.0118	0.0414	0.9936
		0.10	0.0256	0.0122	−0.0115	0.0402	0.9939
		0.15	0.0315	0.0158	0.0139	0.0066	0.9920
		0.20	0.0216	0.0143	−0.0228	0.0877	0.9928
SHAP-PLS	Past	–	0.0321	0.0230	0.0599	0.0372	0.9884
	Comb.	0.00	0.0376	0.0038	0.0675	−0.0634	0.9981
		0.05	0.0389	0.0236	0.0427	−0.0021	0.9881
		0.10	0.0317	0.0194	0.0106	0.0204	0.9903
		0.15	0.0257	0.0050	0.0064	0.0051	0.9975
		0.20	0.0397	0.0200	0.0581	−0.0225	0.9899
