# Supplementary material for: Equity premium forecasting with reliability-screened forward-looking signals
Source: PLoS One. 2026 May 15;21(5):e0341578. doi: 10.1371/journal.pone.0341578 (PMC13178993; doi:10.1371/journal.pone.0341578)
Supplement: S3 Appendix — (PDF) [file pone.0341578.s003.pdf]

S3. Sensitivity of the tail-conditional R^2 diagnostics to alternative quantile levels

The two tables in this appendix extend Table 5 by reporting $R^2_{DOS}(q)$ and $R^2_{UOS}(q)$ over $q \in \{5\%, 10\%, 15\%, 20\%\}$. Formal definitions are given in Section 3.3, so we focus here only on sensitivity to the choice of q. The $q = 10\%$ column reproduces the baseline tail-conditional results reported in the main text. Across the 30 specifications reported in Table 5, the sign of $R^2_{DOS}(q)$ remains unchanged over the full quantile grid for 26 specifications, and the sign of $R^2_{UOS}(q)$ remains unchanged for 22 specifications. Thus, the state-dependent patterns emphasized in Section 5.2 are not driven by the single choice $q = 10\%$. At the same time, the results are not mechanically monotone in q, so q is best viewed as a diagnostic choice rather than as a parameter expected to rank all specifications in one direction.

Table S9: **Conditional downside out-of-sample R^2 by feature family and estimation method.** The table reports out-of-sample R^2 computed within downside market states, defined as test-window months in which the market return falls in the lower q-quantile of its empirical distribution. The reliability threshold τ refers to the minimum individual predictor out-of-sample R^2 from Stage 1 required for its forward-looking forecast to be included in the combined feature set.

Method	Feature	τ	Quantile			
			0.05	0.10	0.15	0.20
–	Past	–	−0.0152	−0.0218	−0.0220	−0.0233
	Comb.	0.00	−0.0055	−0.0149	−0.0122	−0.0128
		0.05	−0.0133	−0.0192	−0.0192	−0.0198
		0.10	−0.0089	−0.0184	−0.0184	−0.0197
		0.15	−0.0222	−0.0323	−0.0292	−0.0299
		0.20	−0.0094	−0.0199	−0.0183	−0.0192
PCA	Past	–	−0.0215	−0.0160	−0.0133	−0.0118
	Comb.	0.00	−0.0162	−0.0186	−0.0172	−0.0190
		0.05	−0.0249	−0.0263	−0.0224	−0.0203
		0.10	−0.0202	−0.0208	−0.0173	−0.0153
		0.15	−0.0207	−0.0190	−0.0168	−0.0141
		0.20	−0.0031	−0.0047	−0.0030	−0.0006
PLS	Past	–	0.0133	−0.0115	−0.0092	−0.0162
	Comb.	0.00	−0.0062	−0.0072	−0.0085	−0.0106
		0.05	0.0545	0.0394	0.0488	0.0493
		0.10	0.0527	0.0386	0.0421	0.0389
		0.15	0.0879	0.0783	0.0849	0.0856
		0.20	0.0129	0.0087	0.0087	0.0047
SHAP-PCA	Past	–	−0.0010	−0.0002	−0.0044	−0.0083
	Comb.	0.00	−0.0009	0.0062	0.0096	0.0083
		0.05	0.0138	0.0127	0.0067	0.0038
		0.10	−0.0302	−0.0355	−0.0244	−0.0229
		0.15	−0.0360	−0.0377	−0.0370	−0.0359
		0.20	−0.0076	−0.0096	−0.0063	−0.0030
SHAP-PLS	Past	–	0.0212	0.0072	−0.0022	−0.0086
	Comb.	0.00	0.0360	0.0276	0.0241	0.0222
		0.05	0.0545	0.0300	0.0517	0.0514
		0.10	0.0352	0.0063	−0.0133	−0.0112
		0.15	0.0175	0.0246	0.0172	0.0179
		0.20	0.0851	0.0734	0.0660	0.0648

The downside results preserve the main qualitative pattern from the baseline analysis. In particular,

PLS-based combined specifications continue to show the clearest downside alignment across the full q-grid. For example, the unscreened PLS Combined specification with $\tau = 0.15$ remains positive in $R^2_{DOS}(q)$ at all four quantiles, while the $\tau = 0.05$ and $\tau = 0.10$ cases are also uniformly positive. By contrast, the raw Combined and unscreened PCA blocks remain mostly weak or negative on the downside, and the SHAP-screened cases are more mixed. This is consistent with the main-text interpretation that supervised low-dimensional representations more often preserve downside-relevant predictive content.

Table S10: **Conditional upside out-of-sample R^2 by feature family and estimation method.** The table reports out-of-sample R^2 computed within upside market states, defined as test-window months in which the market return lies in the upper q-quantile of its empirical distribution. The reliability threshold τ refers to the minimum individual predictor out-of-sample R^2 from Stage 1 required for its forward-looking forecast to be included in the combined feature set.

Method	Feature	τ	Quantile			
			0.05	0.10	0.15	0.20
–	Past	–	−0.0140	−0.0017	0.0010	0.0050
	Comb.	0.00	−0.0283	−0.0227	−0.0258	−0.0242
		0.05	−0.0128	−0.0008	−0.0010	0.0030
		0.10	0.0114	0.0173	0.0171	0.0197
		0.15	0.0126	0.0190	0.0189	0.0203
		0.20	−0.0108	0.0015	0.0011	0.0034
PCA	Past	–	0.0222	0.0241	0.0152	0.0135
	Comb.	0.00	0.0245	0.0213	0.0234	0.0246
		0.05	0.0318	0.0374	0.0329	0.0308
		0.10	0.0306	0.0314	0.0281	0.0249
		0.15	−0.0005	0.0095	0.0036	0.0044
		0.20	0.0067	0.0093	0.0046	0.0033
PLS	Past	–	−0.0372	0.0029	0.0120	0.0268
	Comb.	0.00	0.0033	0.0055	0.0012	−0.0006
		0.05	−0.0574	−0.0457	−0.0525	−0.0361
		0.10	−0.0641	−0.0525	−0.0541	−0.0347
		0.15	−0.1033	−0.0887	−0.1010	−0.0804
		0.20	−0.0077	−0.0110	−0.0147	−0.0107
SHAP-PCA	Past	–	−0.0223	−0.0033	0.0025	0.0101
	Comb.	0.00	0.0265	0.0236	0.0236	0.0246
		0.05	0.0003	0.0087	0.0106	0.0148
		0.10	0.0251	0.0293	0.0388	0.0440
		0.15	0.0604	0.0589	0.0614	0.0634
		0.20	0.0011	0.0063	0.0070	0.0102
SHAP-PLS	Past	–	−0.0037	0.0212	0.0259	0.0364
	Comb.	0.00	−0.0213	−0.0241	−0.0308	−0.0289
		0.05	−0.0147	−0.0361	−0.0341	−0.0173
		0.10	0.0147	0.0478	0.0569	0.0726
		0.15	−0.0242	−0.0261	−0.0284	−0.0255
		0.20	−0.1095	−0.0593	−0.0472	−0.0291

The upside results reinforce the complementary tilt discussed in the main text. PCA-based specifications remain the most consistently positive in $R^2_{UOS}(q)$, and the SHAP-PCA Combined specification with $\tau = 0.15$ provides a particularly stable example, remaining positive at all four quantiles. By contrast, the PLS Combined specification with $\tau = 0.15$—which performs best on the downside—remains negative in $R^2_{UOS}(q)$ throughout the full q-grid. Thus, the broad contrast between PCA-type upside participation and

PLS-type downside alignment is not an artifact of focusing only on the 10% tail.

Taken together, the additional quantile analysis supports the same interpretation as in the main text. Changing q mainly affects the sharpness of the diagnostic rather than its economic meaning: Narrower tails isolate more extreme episodes and therefore produce more volatile conditional R^2 values, whereas broader tails smooth the statistics by including more moderately adverse or favorable months. We therefore retain $q = 10\%$ in the main text as a balanced benchmark between tail focus and tail sample size, while reporting the full quantile sensitivity here.
