# Supplementary material for: Equity premium forecasting with reliability-screened forward-looking signals
Source: PLoS One. 2026 May 15;21(5):e0341578. doi: 10.1371/journal.pone.0341578 (PMC13178993; doi:10.1371/journal.pone.0341578)
Supplement: S4 Appendix — (PDF) [file pone.0341578.s004.pdf]

S4. Robustness of portfolio performance to transaction costs and alternative volatility estimators

Portfolio performance net of transaction costs. This appendix provides additional robustness checks for the portfolio evaluation in Section 5.2. Whereas Appendix S2 examines whether the forecasting results are robust to changes in the learning algorithm and equity universe, the present appendix focuses on the robustness of the economic evaluation to portfolio implementation choices. Specifically, we consider the role of transaction costs and the volatility estimator used for portfolio scaling.

Table S11: **Net of transaction cost portfolio performance by feature family and estimation method.** All entries are computed after deducting proportional transaction costs of 25 basis points per unit of turnover. The table reports Sharpe ratio, Sortino ratio, certainty equivalent return (CER) with risk aversion $\gamma = 3$, maximum drawdown (MDD), and turnover for various forecasting strategies. The reliability threshold τ refers to the minimum individual predictor out-of-sample R^2 from Stage 1 required for its forward-looking forecast to be included in the combined feature set.

Method	Feature	τ	Sharpe	Sortino	CER	MDD	Turnover
Buy & Hold			0.4560	0.6626	0.0347	0.5022	–
CAPM	Past	–	0.2446	0.3276	0.0041	0.5167	2.7710
FF3			0.1783	0.2441	−0.0109	0.5964	5.3486
–	Past	–	0.2593	0.3426	0.0028	0.5920	0.8173
	Comb.	0.00	0.1668	0.2160	−0.0102	0.5561	0.7960
		0.05	0.2676	0.3552	0.0042	0.5545	0.8026
		0.10	0.3392	0.4622	0.0152	0.5471	0.7645
		0.15	0.2771	0.3705	0.0046	0.5892	0.9704
		0.20	0.2480	0.3274	0.0012	0.5495	0.9298
PCA	Past	–	0.3077	0.4292	0.0091	0.6645	0.6818
	Comb.	0.00	0.3679	0.5077	0.0202	0.5596	1.0121
		0.05	0.3235	0.4533	0.0112	0.6632	0.7619
		0.10	0.3127	0.4373	0.0095	0.6642	0.6648
		0.15	0.2343	0.3116	−0.0018	0.6645	0.7151
		0.20	0.2833	0.3819	0.0066	0.6645	0.5879
PLS	Past	–	0.4861	0.6915	0.0393	0.6564	1.7927
	Comb.	0.00	0.2458	0.3276	0.0009	0.5464	1.6234
		0.05	0.3979	0.5534	0.0262	0.4794	2.6328
		0.10	0.4525	0.6316	0.0341	0.5492	2.5089
		0.15	0.4972	0.7404	0.0395	0.4674	2.8478
		0.20	0.3251	0.4524	0.0155	0.4045	1.5598
SHAP-PCA	Past	–	0.4103	0.5603	0.0272	0.5414	1.4063
	Comb.	0.00	0.3919	0.5598	0.0236	0.4971	1.5568
		0.05	0.4750	0.6945	0.0375	0.3542	1.5452
		0.10	0.4498	0.6565	0.0329	0.4695	1.8563
		0.15	0.3658	0.5127	0.0168	0.6645	0.7999
		0.20	0.3265	0.4474	0.0136	0.6518	0.8672
SHAP-PLS	Past	–	0.5214	0.7631	0.0450	0.4016	2.0991
	Comb.	0.00	0.4197	0.6229	0.0292	0.3795	1.7891
		0.05	0.5984	0.9253	0.0559	0.3709	2.4373
		0.10	0.5653	0.8679	0.0530	0.4612	1.8675
		0.15	0.2960	0.4027	0.0093	0.5345	1.5556
		0.20	0.6365	1.0290	0.0588	0.3337	2.5964

Table S11 extends the main text net of transaction cost results to the full admission threshold grid

$\tau \in \{0.00, 0.05, 0.10, 0.15, 0.20\}$ under the baseline assumption of 25 basis points per unit of turnover, while Table S12 reports additional sensitivity checks at the representative interior threshold $\tau = 0.10$ under alternative cost assumptions of 10 and 50 basis points. As expected, higher transaction costs mechanically attenuate net portfolio performance, particularly for specifications with relatively high turnover. Even so, the main qualitative conclusions remain intact. At $\tau = 0.10$, the Combined specification continues to outperform the Past benchmark in the raw, PCA, SHAP-PCA, and SHAP-PLS blocks under both 10 and 50 basis points, whereas the PLS block remains more sensitive to trading frictions. More broadly, several Combined specifications continue to retain economically meaningful gains after transaction costs, especially when the forecasting inputs are organized through supervised low-dimensional representations. Thus, the economic value documented in the main text is not driven solely by ignoring trading frictions.

Table S12: **Net of transaction cost portfolio performance using Random Forest ($\tau = 0.10$), by feature family and estimation method.** Panel A reports results after deducting proportional transaction costs of 10 basis points per unit of turnover. Panel B reports results for 50 basis points. The table reports Sharpe ratio, Sortino ratio, certainty equivalent return (CER) with risk aversion $\gamma = 3$, and maximum drawdown (MDD).

Panel A: Net of 10 bps					
Method	Feature	Sharpe	Sortino	CER	MDD
CAPM	Past	0.2731	0.3679	0.0082	0.5143
FF3		0.2273	0.3137	−0.0029	0.5947
–	Past	0.2668	0.3530	0.0040	0.5913
	Comb.	0.3461	0.4721	0.0164	0.5470
PCA	Past	0.3137	0.4379	0.0101	0.6645
	Comb.	0.3185	0.4458	0.0105	0.6642
PLS	Past	0.5014	0.7147	0.0419	0.6562
	Comb.	0.4773	0.6684	0.0378	0.5471
SHAP-PCA	Past	0.4235	0.5794	0.0293	0.5411
	Comb.	0.4663	0.6822	0.0358	0.4639
SHAP-PLS	Past	0.5412	0.7950	0.0481	0.3945
	Comb.	0.5816	0.8949	0.0559	0.4504
Panel B: Net of 50 bps					
Method	Feature	Sharpe	Sortino	CER	MDD
CAPM	Past	0.1969	0.2613	−0.0027	0.5208
FF3		0.0966	0.1306	−0.0243	0.6237
–	Past	0.2466	0.3254	0.0007	0.5931
	Comb.	0.3277	0.4457	0.0133	0.5474
PCA	Past	0.2978	0.4149	0.0074	0.6645
	Comb.	0.3030	0.4232	0.0078	0.6642
PLS	Past	0.4603	0.6527	0.0349	0.6566
	Comb.	0.4109	0.5705	0.0279	0.5560
SHAP-PCA	Past	0.3884	0.5286	0.0237	0.5418
	Comb.	0.4223	0.6139	0.0282	0.4804
SHAP-PLS	Past	0.4883	0.7103	0.0397	0.4133
	Comb.	0.5381	0.8230	0.0482	0.4789

Alternative volatility estimators for portfolio scaling. We next examine whether the portfolio results depend on the particular variance estimator used in the allocation rule. In the baseline implementation, portfolio weights are scaled using a backward-looking variance estimate from a trailing 5-year window. As an alternative, we construct recursive one-step-ahead conditional variance forecasts for the risky-return innovation series,

$$\varepsilon_{t+1} = r_{t+1} - \hat{r}_{t+1|t},$$

using four GARCH-family models. GARCH(1,1), GJR-GARCH(1,1), EGARCH(1,1), and APARCH(1,1). The GJR-GARCH and EGARCH specifications capture asymmetric volatility responses to positive and negative return innovations. The APARCH model nests a flexible power parameter δ in the conditional variance recursion, where δ is freely estimated by maximum likelihood rather than fixed a priori. Following Patton (2011), forecast accuracy is evaluated using the quasi-likelihood (QLIKE) loss computed period by period and then averaged across the out-of-sample evaluation window,

$$\bar{Q} = \frac{1}{T}\sum_{t=1}^{T}\left(\frac{\mathrm{RV}_t}{\hat{\sigma}_t^2} - \ln\frac{\mathrm{RV}_t}{\hat{\sigma}_t^2} - 1\right),$$

where RV_t is proxied by the squared one-step-ahead return innovation ε_t^2. Lower values indicate better conditional variance forecasts.

Table S13 shows a clear and uniform pattern: EGARCH delivers the lowest average out-of-sample QLIKE loss in every reported specification. This dominance holds across both Past and Combined feature sets and across all representation choices. The result suggests that allowing for asymmetric and exponential volatility dynamics provides the most accurate conditional variance forecasts for the return-innovation series generated by the forecasting pipeline. We therefore use EGARCH as the alternative volatility estimator in the portfolio scaling robustness exercise reported in Table S14.

Table S14 allows a direct comparison with the main-text portfolio results based on the trailing 5-year rolling variance estimator. Relative to that baseline, replacing the historical variance proxy with the recursively updated EGARCH forecast generally increases turnover, indicating more responsive portfolio rebalancing. At the same time, however, the additional trading intensity is typically accompanied by stronger economic performance rather than by turnover alone. For example, at $\tau = 0.10$, the raw Combined specification improves from 0.3507 to 0.3945 in Sharpe ratio while reducing maximum drawdown from 0.5469 to 0.4304; the PLS Combined specification improves from 0.4937 to 0.6342 in Sharpe ratio and from 0.0403 to 0.0593 in CER while lowering maximum drawdown from 0.5457 to 0.3666; and the SHAP-PLS Combined specification rises from 0.5924 to 0.6386 in Sharpe ratio while reducing maximum drawdown from 0.4449 to 0.3369. These gains occur alongside higher turnover, suggesting that the EGARCH-based variance forecast induces more active rebalancing, but that this greater responsiveness translates into economically meaningful improvements in risk-adjusted performance and downside protection in many of the stronger specifications. More broadly, the leading specifications under the baseline variance rule continue to rank highly under EGARCH-based scaling, so the portfolio conclusions are not an artifact of the trailing-window variance estimator.

References

Patton, A. J. (2011). Volatility forecast comparison using imperfect volatility proxies. *Journal of econometrics*, 160(1):246–256.

Table S13: **Average quasi-likelihood (QLIKE) loss for conditional variance forecasts by feature family, estimation method, and volatility specification.** Lower values indicate better variance forecasts. The reliability threshold τ refers to the minimum individual predictor out-of-sample R^2 from Stage 1 required for its forward-looking forecast to be included in the combined feature set.

Method	Feature	τ	GARCH	GJR	APARCH	EGARCH
–	Past	–	1.3712	1.3451	1.3551	1.3391
	Comb.	0.00	1.4037	1.3659	1.3789	1.3598
		0.05	1.3772	1.3581	1.3685	1.3442
		0.10	1.3612	1.3355	1.3463	1.3305
		0.15	1.3514	1.3264	1.3377	1.3162
		0.20	1.3968	1.3751	1.3806	1.3640
PCA	Past	–	1.2939	1.2725	1.2763	1.2558
	Comb.	0.00	1.3528	1.3343	1.3377	1.3155
		0.05	1.3118	1.2970	1.3055	1.2850
		0.10	1.3397	1.3216	1.3358	1.3080
		0.15	1.3045	1.2831	1.2901	1.2651
		0.20	1.2912	1.2705	1.2812	1.2582
PLS	Past	–	1.4709	1.4577	1.4583	1.4477
	Comb.	0.00	1.3693	1.3379	1.3498	1.3330
		0.05	1.3257	1.3075	1.3187	1.2888
		0.10	1.3757	1.3610	1.3687	1.3477
		0.15	1.3681	1.3466	1.3526	1.3369
		0.20	1.3772	1.3558	1.3667	1.3451
SHAP-PCA	Past	–	1.3865	1.3739	1.3687	1.3564
	Comb.	0.00	1.3803	1.3570	1.3663	1.3472
		0.05	1.3273	1.3126	1.3246	1.2944
		0.10	1.4082	1.4010	1.4029	1.3868
		0.15	1.3250	1.3179	1.3125	1.2943
		0.20	1.3251	1.3053	1.3090	1.2859
SHAP-PLS	Past	–	1.4270	1.4103	1.4282	1.3986
	Comb.	0.00	1.3460	1.3343	1.3468	1.3203
		0.05	1.4330	1.4174	1.4379	1.4086
		0.10	1.4214	1.3884	1.4050	1.3883
		0.15	1.3723	1.3547	1.3563	1.3382
		0.20	1.2417	1.2243	1.2272	1.2151

Table S14: **Portfolio performance with EGARCH-based volatility, by feature family and estimation method.** The mean–variance portfolio uses conditional variance estimated via an EGARCH(1,1) model in place of the rolling-window estimator. The table reports Sharpe ratio, Sortino ratio, certainty equivalent return (CER) with risk aversion $\gamma = 3$, maximum drawdown (MDD), and turnover for various forecasting strategies. The reliability threshold τ refers to the minimum individual predictor out-of-sample R^2 from Stage 1 required for its forward-looking forecast to be included in the combined feature set.

Method	Feature	τ	Sharpe	Sortino	CER	MDD	Turnover
Buy & Hold			0.4560	0.6626	0.0347	0.5022	–
CAPM	Past	–	0.3705	0.5102	0.0228	0.3771	2.6284
FF3			0.3068	0.4216	0.0128	0.4387	5.3559
–	Past	–	0.3614	0.4964	0.0203	0.4521	1.8827
	Comb.	0.00	0.2864	0.3862	0.0095	0.4283	1.8249
		0.05	0.3697	0.5094	0.0214	0.4220	1.8371
		0.10	0.3945	0.5471	0.0252	0.4304	1.7912
		0.15	0.3589	0.4920	0.0193	0.4588	1.9920
		0.20	0.3532	0.4842	0.0189	0.4562	2.0019
PCA	Past	–	0.3756	0.5242	0.0229	0.4487	1.9360
	Comb.	0.00	0.4205	0.5868	0.0292	0.4270	1.7241
		0.05	0.3995	0.5628	0.0260	0.4612	2.1844
		0.10	0.3851	0.5380	0.0242	0.4357	2.0421
		0.15	0.3854	0.5418	0.0243	0.4249	1.9931
		0.20	0.4146	0.5840	0.0286	0.3709	1.9530
PLS	Past	–	0.6126	0.9183	0.0584	0.4738	2.1826
	Comb.	0.00	0.3693	0.5114	0.0217	0.5055	2.5174
		0.05	0.6115	0.9389	0.0551	0.3085	3.1339
		0.10	0.6342	0.9809	0.0593	0.3666	2.8576
		0.15	0.6871	1.1241	0.0630	0.3115	3.1684
		0.20	0.3942	0.5512	0.0257	0.4882	2.2808
SHAP-PCA	Past	–	0.5119	0.7298	0.0432	0.3742	2.2920
	Comb.	0.00	0.5016	0.7218	0.0417	0.4344	2.5168
		0.05	0.5125	0.7359	0.0430	0.4069	2.3479
		0.10	0.4997	0.7175	0.0416	0.4701	2.4949
		0.15	0.4636	0.6635	0.0358	0.4566	2.0646
		0.20	0.4419	0.6297	0.0325	0.4140	1.8664
SHAP-PLS	Past	–	0.6221	0.9445	0.0606	0.3207	2.4148
	Comb.	0.00	0.5310	0.7822	0.0448	0.3418	2.4329
		0.05	0.6867	1.0691	0.0679	0.3678	2.9734
		0.10	0.6386	1.0030	0.0633	0.3369	2.5445
		0.15	0.4410	0.6253	0.0324	0.3938	2.3297
		0.20	0.7309	1.1963	0.0718	0.3242	2.9111
