# Supplementary material for: Equity premium forecasting with reliability-screened forward-looking signals
Source: PLoS One. 2026 May 15;21(5):e0341578. doi: 10.1371/journal.pone.0341578 (PMC13178993; doi:10.1371/journal.pone.0341578)
Supplement: S5 Appendix — (PDF) [file pone.0341578.s005.pdf]

S5. Full sensitivity of the sigma ablation study

Tables S15 and S16 extend the sigma ablation in Section 5.3.2 by reporting results over the full admission-threshold grid, $\tau \in \{0.0, 0.05, 0.1, 0.15, 0.2\}$. The purpose of this appendix is not to select an ex post optimal threshold, but to show how the marginal contribution of the Stage 1 uncertainty proxy varies with the selectivity of forward-looking signal admission. As in the main text, we place the greatest weight on the non-SHAP blocks as the cleanest comparison of the role of $\hat{\sigma}_{k,t+1|t}$, while the SHAP-screened results are interpreted as complementary sensitivity evidence.

Prediction metrics. Table S15 broadly reinforces the main text interpretation from Table 7. First, the contribution of $\hat{\sigma}_{k,t+1|t}$ is clearly non-monotonic in τ, which supports our broader interpretation of the admission threshold as a selectivity device rather than as a parameter expected to rank performance in a single direction. In the unreduced combined specification, sigma inclusion is mildly harmful at very loose admission levels but becomes beneficial at interior thresholds, with the clearest gains appearing at $\tau = 0.10$ and $\tau = 0.15$. Importantly, these gains are tilted toward downside states: ΔR^2_{DOS} is positive across all thresholds in the raw block, whereas ΔR^2_{UOS} is smaller and often negative. Thus, even when the aggregate change in R^2_{OS} is modest, the uncertainty proxy continues to improve the model's alignment with adverse market states.

Second, the full threshold grid confirms that the PCA block remains essentially flat. Across all τ values, the changes in R^2 -based metrics and RRMSE are close to zero, indicating that once the combined signal is compressed along variance-maximizing directions, the uncertainty channel contributes little additional forecasting content. By contrast, the PLS block again provides the clearest non-SHAP evidence for the incremental value of $\hat{\sigma}_{k,t+1|t}$. For $\tau = 0.05$, 0.10 and 0.15, sigma inclusion improves aggregate out-of-sample fit and lowers RRMSE, while the gain in downside conditional accuracy is especially pronounced. For example, ΔR^2_{OS} reaches 0.0164 at $\tau = 0.15$, alongside $\Delta R^2_{DOS} = 0.0683$. This pattern is consistent with the interpretation advanced in the main text: The uncertainty proxy is not simply a rescaling of the conditional mean forecast, but a state-dependent reliability signal whose value becomes most visible in supervised low-dimensional representations.

The SHAP-screened results remain informative but should be interpreted more cautiously. At some thresholds, especially under SHAP-PCA at $\tau = 0.00$ and SHAP-PLS at $\tau = 0.05$, sigma inclusion delivers sizeable improvements in aggregate out-of-sample fit. However, these gains are also more threshold-sensitive, and in some cases the tail asymmetry becomes extreme under tight admission. The full grid therefore supports the main-text choice to treat SHAP-based ablation results as supplementary robustness evidence rather than as the cleanest estimate of sigma's marginal contribution.

Portfolio performance. Table S16 shows that the economic consequences of the sigma ablation are also threshold-dependent. In the raw combined specification, sigma inclusion weakens gross portfolio performance at $\tau = 0.00$ and $\tau = 0.05$, but becomes beneficial at the representative interior thresholds emphasized in the main text. In particular, both $\tau = 0.10$ and $\tau = 0.15$ deliver positive changes in Sharpe, Sortino, and CER together with lower maximum drawdown. This mirrors the prediction-side evidence that the uncertainty proxy becomes more useful once admission is sufficiently selective to exclude weaker Stage 1 signals.

The PCA block again remains economically negligible throughout the full grid, which is consistent with its near-zero statistical contribution in Table S16. By contrast, the PLS block once more provides the strongest non-SHAP support for the uncertainty channel. At $\tau = 0.10$ and $\tau = 0.15$, sigma inclusion raises the Sharpe ratio, the Sortino ratio, and the certainty equivalent return, while also reducing maximum drawdown. The largest gains occur at $\tau = 0.15$, where the increases in Sharpe and Sortino are especially pronounced. These improvements, however, are accompanied by higher turnover, indicating that the economic gains are not driven by mechanically smoother positions or reduced trading intensity. Rather, they appear to reflect a more informative signal entering the allocation rule.

The SHAP-screened portfolio results again show that larger gains are possible in some specifications, but they also underscore the same threshold sensitivity seen on the prediction side. SHAP-PCA performs

Table S15: **Full-threshold sensitivity of the sigma ablation: Out-of-sample prediction metrics for the combined feature family.** Throughout the table, Δ denotes the difference in performance between the specification that includes the Stage 1 uncertainty proxy $\hat{\sigma}_{k,t+1|t}$ and the corresponding specification that excludes it. ΔR^2_{IS}, ΔR^2_{OS}, ΔR^2_{DOS}, ΔR^2_{UOS}, and ΔRRMSE over the admission-threshold grid τ, where τ is the minimum individual Stage 1 out-of-sample R^2 required for a predictor's forward-looking signals to enter the combined set. Positive values of ΔR^2_{IS}, ΔR^2_{OS}, ΔR^2_{DOS}, and ΔR^2_{UOS} indicate better performance with $\hat{\sigma}_{k,t+1|t}$, whereas negative values of ΔRRMSE indicate improvement because forecast error is reduced.

Method	τ	ΔR^2_{IS}	ΔR^2_{OS}	ΔR^2_{DOS}	ΔR^2_{UOS}	ΔRRMSE
	0.00	0.0022	−0.0084	0.0070	−0.0277	0.0041
	0.05	0.0021	−0.0017	0.0083	−0.0151	0.0008
–	0.10	−0.0003	0.0058	0.0197	−0.0065	−0.0029
	0.15	0.0021	0.0035	0.0047	0.0024	−0.0017
	0.20	0.0012	−0.0008	0.0056	−0.0058	0.0003
	0.00	0.0000	0.0001	0.0000	−0.0001	0.0000
	0.05	0.0000	0.0000	0.0000	0.0001	0.0000
PCA	0.10	0.0000	−0.0001	−0.0002	−0.0002	0.0000
	0.15	0.0000	0.0000	−0.0002	0.0006	0.0000
	0.20	0.0000	0.0008	0.0000	0.0026	−0.0004
	0.00	−0.0001	−0.0025	0.0004	−0.0003	0.0012
	0.05	0.0082	0.0128	0.0633	−0.0511	−0.0064
PLS	0.10	0.0084	0.0136	0.0611	−0.0414	−0.0068
	0.15	0.0066	0.0164	0.0683	−0.0452	−0.0082
	0.20	−0.0016	−0.0019	0.0271	−0.0403	0.0009
	0.00	0.0004	0.0382	0.0430	0.0413	−0.0191
	0.05	0.0033	0.0159	0.0416	−0.0059	−0.0079
SHAP-PCA	0.10	0.0047	0.0103	0.0171	−0.0324	−0.0052
	0.15	−0.0019	−0.0027	−0.0021	−0.0051	0.0013
	0.20	0.0015	0.0030	0.0069	−0.0070	−0.0015
	0.00	0.0002	0.0066	0.0336	−0.0225	−0.0033
	0.05	0.0006	0.0303	0.0446	−0.0110	−0.0152
SHAP-PLS	0.10	0.0045	0.0143	0.0314	−0.0301	−0.0072
	0.15	−0.0045	−0.0036	0.0382	−0.0433	0.0018
	0.20	0.0007	−0.0120	0.1080	−0.1609	0.0060

strongly at loose-to-moderate thresholds, whereas SHAP-PLS is favorable at $\tau = 0.05$ and $\tau = 0.10$ but deteriorates sharply at $\tau = 0.15$.

In summary, Tables S15 and S16 reinforce the central message of Section 5.3.2: the Stage 1 uncertainty proxy has a meaningful incremental role, but its value depends on how selectively forward-looking signals are admitted and how the resulting combined pool is represented before entering Stage 2. Across the full threshold grid, the most stable evidence continues to come from the non-SHAP specifications, where the benefits of sigma are concentrated in downside-state forecasting and, under PLS, carry through into economically meaningful improvements in gross portfolio performance.

Table S16: **Full-threshold sensitivity of the sigma ablation: Portfolio performance for the combined feature family.** Throughout the table, Δ denotes the difference in performance between the specification that includes the Stage 1 uncertainty proxy $\hat{\sigma}_{k,t+1|t}$ and the corresponding specification that excludes it. The table reports ΔSharpe, ΔSortino, ΔCER, ΔMDD and ΔTurnover over the admission-threshold grid τ, where τ is the minimum individual Stage 1 out-of-sample R^2 required for a predictor's forward-looking signals to enter the combined set. CER denotes the certainty equivalent return with risk aversion $\gamma = 3$. Positive values of ΔSharpe, ΔSortino, and ΔCER indicate better performance with $\hat{\sigma}_{k,t+1|t}$, whereas negative values of ΔMDD indicate improvement because maximum drawdown is reduced. ΔTurnover is reported for transparency and does not by itself imply improvement or deterioration.

Method	τ	ΔSharpe	ΔSortino	ΔCER	ΔMDD	ΔTurnover
	0.00	−0.0930	−0.1282	−0.0133	−0.0463	−0.0406
	0.05	−0.0587	−0.0934	−0.0082	0.0000	0.0648
–	0.10	0.0415	0.0614	0.0080	−0.0817	−0.0003
	0.15	0.0316	0.0465	0.0052	−0.0488	0.1172
	0.20	−0.0190	−0.0273	−0.0029	−0.0474	0.0631
	0.00	0.0013	0.0022	0.0002	−0.0026	0.0083
	0.05	0.0003	0.0005	0.0000	0.0000	−0.0001
PCA	0.10	−0.0016	−0.0026	−0.0003	0.0000	−0.0007
	0.15	−0.0011	−0.0015	−0.0002	0.0000	0.0003
	0.20	0.0109	0.0171	0.0015	0.0000	−0.0310
	0.00	−0.0417	−0.0698	−0.0078	0.0767	0.1147
	0.05	0.0080	0.0184	0.0014	−0.1045	1.2452
PLS	0.10	0.1017	0.1566	0.0157	−0.0230	1.1540
	0.15	0.1613	0.2832	0.0221	−0.0794	1.4041
	0.20	0.0245	0.0449	0.0068	−0.1714	0.2597
	0.00	0.2222	0.3414	0.0367	−0.1789	−0.5510
	0.05	0.1936	0.3184	0.0322	−0.2021	0.0356
SHAP-PCA	0.10	0.1546	0.2443	0.0302	−0.2130	0.3397
	0.15	−0.0240	−0.0395	−0.0047	0.0285	−0.0710
	0.20	−0.0020	−0.0003	0.0002	−0.0136	0.2643
	0.00	0.1242	0.2136	0.0210	−0.1886	0.4333
	0.05	0.2270	0.4140	0.0347	−0.1349	0.7713
SHAP-PLS	0.10	0.1155	0.2044	0.0206	−0.1290	−0.0448
	0.15	−0.1058	−0.1843	−0.0165	0.1097	−0.2546
	0.20	0.0735	0.1715	0.0045	−0.2203	0.7807
