# Supplementary material for: Equity premium forecasting with reliability-screened forward-looking signals
Source: PLoS One. 2026 May 15;21(5):e0341578. doi: 10.1371/journal.pone.0341578 (PMC13178993; doi:10.1371/journal.pone.0341578)
Supplement: S6 Appendix — (PDF) [file pone.0341578.s006.pdf]

S6. Time variation in SHAP-ranked predictor bundles

For a concrete and representative illustration of the SHAP screening step, this appendix focuses on the SHAP-PLS specification with top-10 screening and the interior reliability threshold $\tau = 0.10$. This choice aligns with the main Stage 2 design, where SHAP screening is applied before dimension reduction under annual expanding window refits, and it also corresponds to one of the more favorable SHAP-PLS cases in the portfolio results reported in the main text and supporting robustness checks.

To keep the stability analysis aligned with the structure of the forecasting pipeline, we work at the predictor-bundle level. Recall that each original predictor enters Stage 2 through up to three related components: the contemporaneous predictor itself, its one-step-ahead conditional mean forecast, and its one-step-ahead forecast-uncertainty proxy. For a given predictor k and year t, we therefore define bundled SHAP importance as

$$S_{k,t} = \sum_{c \in \{\text{level,pred,std}\}} |\phi_{k,c,t}|,$$

where $\phi_{k,c,t}$ is the mean absolute TreeSHAP value of component c associated with predictor k in year t. This aggregation is useful because it treats the predictor as the economically meaningful unit of screening, while avoiding an artificial separation between the predictor's level, forecasted movement, and forecast uncertainty. We then normalize the bundled scores within each year as

$$\tilde{S}_{k,t} = \frac{S_{k,t}}{\sum_j S_{j,t}},$$

so that the annual magnitudes can be interpreted as relative shares of the selected SHAP frontier. This normalization is used only for comparability across years.

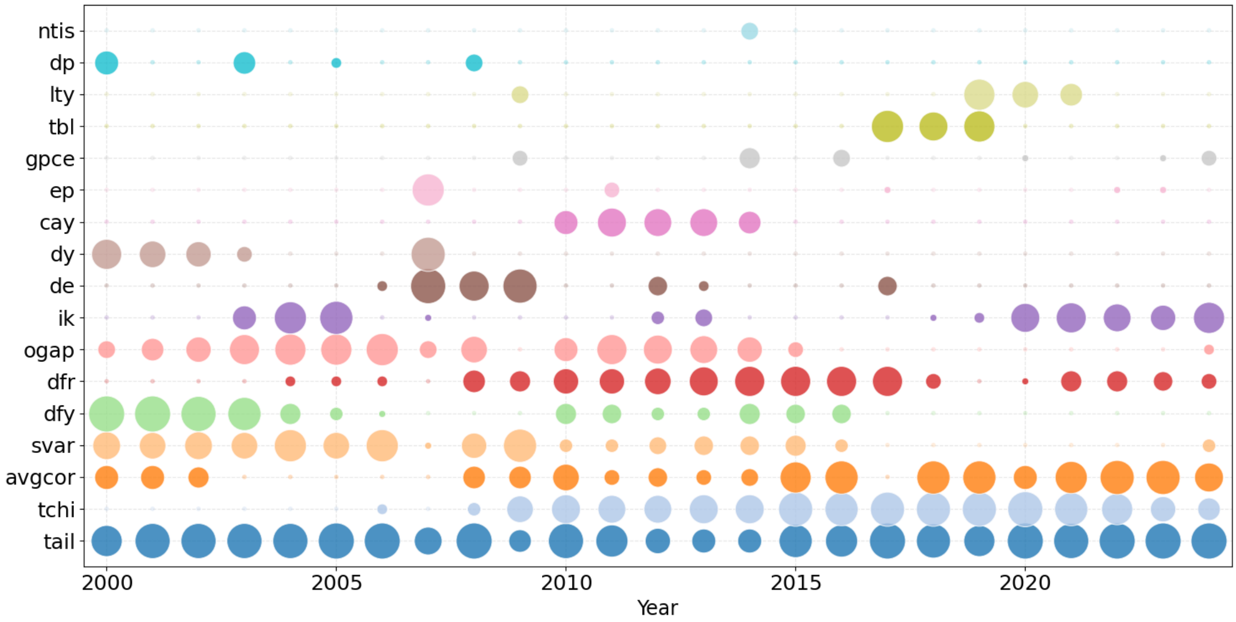

Figure S1: **Time variation in SHAP-ranked predictor bundles.** This figure reports the annual composition of the SHAP-ranked top-10 set for the representative SHAP-PLS specification with $\tau = 0.10$. Each predictor bundle combines the contemporaneous predictor, its one-step-ahead conditional mean forecast, and its forecast-uncertainty proxy. Bubble sizes reflect the bundle's relative SHAP importance within each year.

Figure S1 provides the visual overview. The selected set is not completely reshuffled from year to year. Instead, the figure shows a recurring core in which tail risk remains persistently present, while average

correlation, default-return-spread, the technical composite index, stock market variance, and the output gap also recur frequently across annual refits. At the same time, other bundles appear more episodically, which is consistent with the broader idea of regime-dependent predictability developed in the main text. In other words, the SHAP screen appears to adapt to changing episodes without becoming fully unstable.

This pattern is also economically suggestive. The bundles that recur most often are tilted toward market stress, comovement, credit conditions, and selected macro-financial states. That is broadly consistent with the role that downside-sensitive information appears to play elsewhere in the paper, especially under supervised low-dimensional representations such as PLS. Put differently, the stability plot suggests that SHAP-PLS is not selecting variables at random from year to year. It repeatedly returns to a set of signals that are plausibly relevant when adverse states, cross-market stress, or changes in financial conditions matter for equity premium forecasting. The pattern is consistent with why the representative SHAP-PLS specification can look attractive on downside-aware investment criteria such as the Sortino ratio while maintaining competitive drawdown control relative to much of the broader specification set.

Table S17 complements the figure by summarizing the same stability patterns numerically. "Count" measures how many annual refits include a given predictor bundle in the SHAP-ranked top-10 set. "Avg. rank" measures how highly that bundle tends to appear within the selected set when it is present. "Avg. share" measures how much of the selected annual SHAP frontier the bundle typically accounts for, conditional on selection, and is reported in percent. For bundle k, the average bundled SHAP share is computed as

$$\text{Avg.share}_k = 100 \times \frac{1}{\text{Count}_k} \sum_{t \in T_k} \tilde{S}_{k,t},$$

where T_k is the set of annual refits in which that bundle is selected.

Table S17: **Stability summary for SHAP-ranked predictor bundles.** This table summarizes how often each predictor bundle appears in the annually selected SHAP top-10 set for the representative SHAP-PLS specification with $\tau = 0.10$. A predictor bundle is formed by aggregating the contemporaneous predictor, its one-step-ahead mean forecast, and its forecast-uncertainty proxy into a single bundle. Count reports the number of annual refits, out of 25, in which the bundle appears at least once in the selected top-10 set. Avg. rank is the average within-year rank of the bundle, conditional on selection, after recomputing annual ranks using bundled SHAP importance. Avg. share is the average within-year bundled SHAP share, conditional on selection, reported in percent.

Bundle	Count	Avg. rank	Avg. share (%)
tail	25	2.08	23.4
avgcor	19	4.53	13.8
dfr	19	4.63	9.9
tchi	18	2.78	16.1
svar	18	5.22	11.0
ogap	16	4.25	11.6
dfy	14	5.07	13.8
ik	13	4.77	11.5
de	7	4.29	13.8
gpce	6	6.33	7.8
cay	5	3.60	11.3
dy	5	4.20	13.1
ep	5	6.00	9.6
lty	4	5.25	11.1
dp	4	6.25	8.8
tbl	3	3.67	15.5
ntis	1	9.00	8.5

Taken together, Figure S1 and Table S17 lead to a clear conclusion. The SHAP-ranked top-10 set in the representative SHAP-PLS specification is not perfectly fixed, but neither is it unstable in a way that would negate the parsimony benefit of screening. Instead, a recurring group of predictor bundles remains visible

across annual refits, while their relative importance shifts with the market environment. This is precisely the kind of pattern one would expect in a setting where predictability is state dependent: the dominant economic channel evolves across episodes, but it does so around a persistent core rather than through a complete annual replacement of the selected set.
